# The *LHT* Gene Family in Rice: Molecular Characterization, Transport Functions and Expression Analysis

**DOI:** 10.3390/plants12040817

**Published:** 2023-02-12

**Authors:** Tian Fan, Chunting Wu, Weiqi Yang, Tianxiao Lv, Yuping Zhou, Changen Tian

**Affiliations:** Guangdong Key Laboratory of Plant Adaptation and Molecular Design, Guangzhou Key Laboratory for Functional Study on Plant Stress-Resistant Genes, School of Life Sciences, Guangzhou University, Guangzhou 510006, China

**Keywords:** *LHT* genes, rice, expression pattern, amino acids, transport

## Abstract

Amino acid transporters (AATs) are integral membrane proteins and play important roles in plant growth and development as well as environmental responses. In contrast to the amino acid permease (AAP) subfamily, functional studies of the lysine and histidine transporter (LHT) subfamily have not been made in rice. In the current study, six *LHT* genes were found in the rice genome. To further investigate the functions of these genes, analyses were performed regarding gene and protein structures, chromosomal locations, evolutionary relationships, cis-acting elements of promoters, gene expression, and yeast complementation. We found that the six *OsLHT* genes are distributed on 4 out of the 12 chromosomes and that the six *OsLHT* genes were grouped into two clusters based on the phylogenetic analysis. Protein structure analyses showed that each OsLHT protein has 11 helical transmembrane domains. Yeast complementation assays showed that these Os*LHT* genes have conserved transport substrates within each cluster. The four members from cluster 1 showed broad amino acid selectivity, while OsLHT5 and OsLHT6 may transport other substrates besides amino acids. Additionally, quantitative real-time PCR analysis of the six *OsLHT* genes revealed that they have different expression patterns at different developmental stages and in different tissues. It also revealed that some *OsLHT* genes were responsive to PEG, NaCl and cold treatments, indicating their critical roles in abiotic stress response. Our results will be useful for further characterizing the crucial biological functions of rice *LHT* genes.

## 1. Introduction

Nitrogen (N) is an important nutrient for the growth and development of plants. Plants absorb both inorganic nitrogen (ammonium and nitrate) and organic nitrogen (amino acids, peptides and proteins) by plant roots from the soil [1]. For organic nitrogen, amino acids are the primary transport form in most plant species. In order to meet the N requirements of developing sink organs, amino acids produced in leaves or obtained from roots are transported by phloem [2]. In plants, transport proteins often mediate the transport of amino acids. Amino acid transporters (AATs) are integral membrane proteins which are involved in various processes of plant growth and development, such as absorption, long-distance transport, the distribution of amino acids, and responses to pathogen infection and abiotic stresses [3]. 

AATs have been found and identified in a variety of plant species, such as Arabidopsis [4], rice [5], soybean [6], poplar [7], potato [8], wheat [9], among others. The AATs are classified into three main families in plants: APCs (the amino acid-polyamine-choline transporter family), ATFs (the amino acid transporter family, also known as the AAAP family) and the recently discovered UMAMITs (the usually multiple acids move in and out transporter family) [10]. The ATF family contains at least eight subfamilies, such as LHTs (lysine and histidine transporters), AUXs (auxin transporters), ProTs (proline transporters), ATLs (amino acid transporter-like proteins), GATs (c-aminobutyric acid transporters), AAPs (amino acid permeases), ANTs (aromatic and neutral amino acid transporters) and VAATs (vesicular aminergic-associated transporters) [10]. Typically, the members of the AUX subfamily transport auxin rather than amino acids [11]. In addition, the APC family is divided into three subfamilies in plants: CATs (cationic amino acid transporters), ACTs (amino acid/choline transporters) and PHSs (polyamine H+-symporters) [12]. Recently, it was proposed that UMAMITs are members of the nodulin-like gene family [13] and that several of the family’s members play major roles in bidirectional amino acid transport [14]. Among these families, the AAP family has received the most focus in plants. Compared to the AAP family, further functional studies of the LHT family are required. 

In model plants, such as Arabidopsis [15,16,17] and rice [18,19,20], some LHT protein functions have been investigated to date. In Arabidopsis, 10 members named *LHT1* to *LHT10* have been identified. *AtLHT1* is mainly expressed on the surfaces of roots and plays a role in the root absorption of neutral and acidic amino acids and importation into leaf mesophyll cells [16]. Disruption to *AtLHT1* was found to result in the defective uptake of a range of amino acids and, ultimately, decreases in shoot biomass and seed yield [16]. In addition, *AtLHT1* also acts as a negative regulator of disease resistance; its mutation increases disease resistance to a wide range of pathogens in an SA-dependent manner [17]. Besides amino acids, another study showed that *AtLHT1* and *AtLHT2* may transport ACC (1-aminocyclopropane-1-carboxylate), which acts as a biosynthetic precursor of ethylene [21,22]. Recent research has demonstrated that *AtLHT1* can also be responsible for the translocation of pesticide–amino acid conjugates in Arabidopsis [23,24]. *AtLHT6* is highly expressed in the roots and is involved in the uptake of acidic amino acids and alanine at low and high concentrations [25]. However, it is noteworthy that the study of LHT transporters has been focused on Arabidopsis, and our knowledge of LHT family members in rice is still very limited. Zhao et al. [5] identified six *LHT* members in rice by means of a genome-wide survey; only the function of *OsLHT1* has been studied in depth in recent studies. *OsLHT1* is expressed throughout the root, including the stele, root hairs, exodermis, epidermis, cortex cells, endodermis and sclerenchyma, indicating its function in amino acid acquisition and movement into steles [18]. Meanwhile, disruption to *OsLHT1* decreased amino acid levels in xylem sap and inhibited root-to-shoot amino acid translocation [19]. In addition, *OsLHT1* has been demonstrated to be involved in phloem loading and allocation from leaves to panicles. Knockout of *OsLHT1* affected seed storage and decreased amino acid allocations to grains, resulting in lower grain yield and N-utilization efficiency [19].

Thus, in order to understand the significance of the *OsLHT* gene family with respect to plant growth, development and grain quality in rice, we performed systematic identifications and comprehensive characterizations. In this study, we identified the chromosomal locations, physicochemical properties, duplication events, phylogenetic relationships, gene structures and protein three-dimensional structures of the *OsLHT* genes and complemented these investigations with research on subcellular localization, a transport substrate assay, and analysis of the gene expression patterns of all the LHTs in various tissues and under abiotic stress conditions. The goal was to provide a theoretical foundation for further functional analysis and study of the molecular mechanisms of the rice *LHT* genes involved in rice growth, development and stress resistance.

## 2. Results

### 2.1. Identification of the LHT Gene Family in Rice

In this study, name searches with keywords (e.g., Lysine/Histidine transporter) were made to obtain annotated candidates of LHT family members in RAP-DB (http://rapdb.dna.affrc.go.jp/) (accessed on 20 June 2022). These keyword searches resulted in a total of six *LHT* candidates. Next, the six *LHT* candidate sequences were subjected to InterProScan to confirm the presence of IPR013057 domains. Altogether, six *LHTs* were identified in rice. This was consistent with the previous data published for the rice AAT family [5]. Here, we employ the gene nomenclature published by Zhao et al. [5] for rice *LHTs*. Detailed information about the chromosomal location, FL-cDNA, ORF length and gene locus for each *OsLHT* gene and the physicochemical properties of the OsLHT proteins are listed in Table 1. The proteins encoded by *OsLHT* genes had predicted molecular weights of 47.8 to 55.0 kDa and lengths ranging from 444 to 512 amino acids. In addition, the predicted pI values ranged from 8.87 to 9.20, and all were below 10.0. 

The chromosomal locations of the *OsLHT* genes were determined according to the gene starting position in the rice chromosomes. The *OsLHT* genes were randomly distributed on four chromosomes (Figure S1). Both *OsLHT4* and *OsLHT5* were localized on chromosome 4, *OsLHT2* and *OsLHT6* were localized on chromosome 12, while *OsLHT1* and *OsLHT3* were localized on chromosomes 8 and 5, respectively (Figure S1). In order to understand the expansion patterns of the *OsLHT* gene family, analyses of tandem duplication and segmental duplication were performed. Based on the rice segmental duplication database from RGAP and chromosome distribution analysis, we found that no pairs of genes could be allocated to rice segmental duplication blocks. This suggested that tandem duplication and segmental duplication events were not the cause of the *OsLHT* gene family’s expansion. 

### 2.2. Phylogenetic Analysis of the OsLHT Gene Family

In order to further evaluate in detail phylogenetic relationships and molecular evolution, a maximum-likelihood tree was constructed using MEGA X software by aligning full-length LHT amino acid sequences from one non-vascular land plant (*Physcomitrella patens*, 3 proteins), one non-seed vascular plant (*Selaginella moellendorffii*, 8 proteins) and five seed plants, including *Solanum lycopersicum* (6 proteins), *Arabidopsis thaliana* (10 proteins), *Sorghum bicolor* (8 proteins), *Oryza sativa* (6 proteins) and *Medicago truncatula* (7 proteins). 

**Figure 1:**
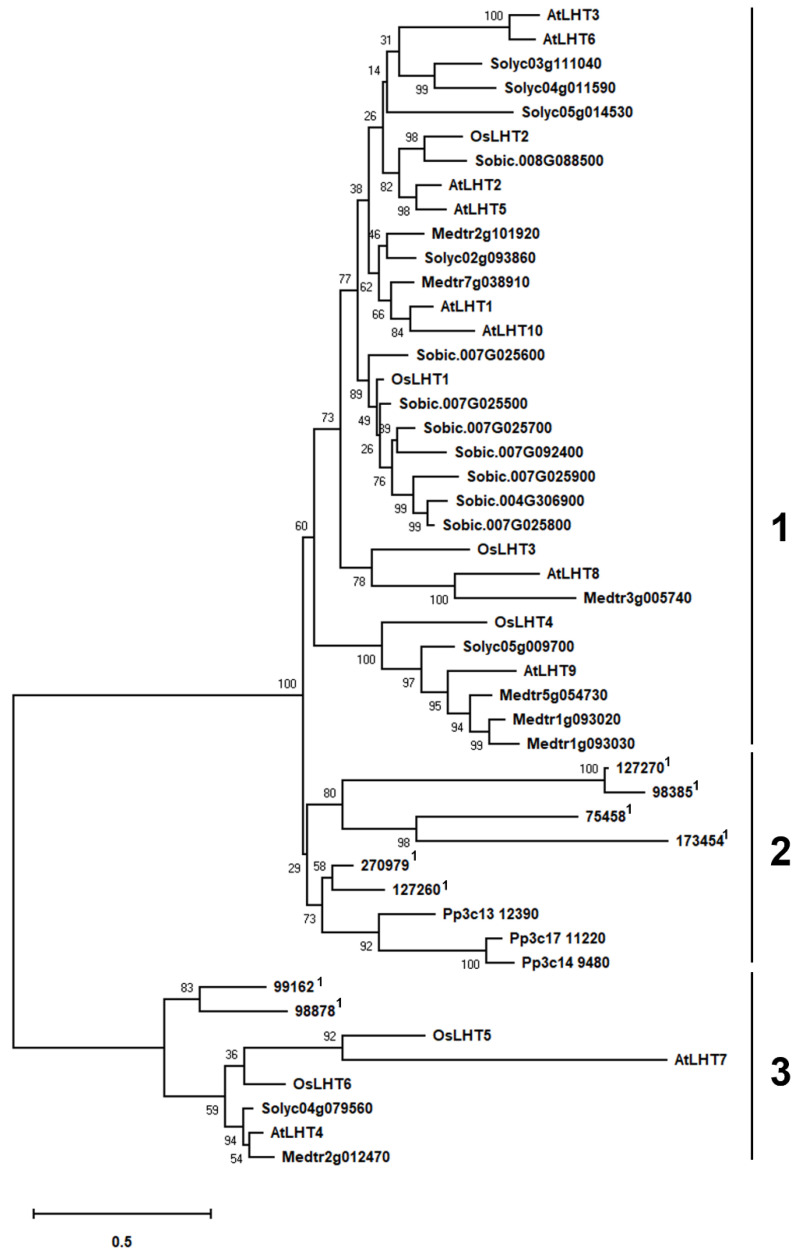
Phylogenetic relationship analysis of LHT proteins from Solanum lycopersicum, Arabidopsis thaliana, Physcomitrella patens, Oryza sativa, Selaginella moellendorffii, Medicago truncatula and Sorghum bicolor. Multiple-protein-sequence alignment was performed using Clustal X (1.83) software, and the tree was constructed according to the maximum-likelihood (ML) method with the *p*-distance substitution model using MEGA X software. We used 1000 replicates in the bootstrap analysis to determine the support value for each branch. The clusters are numbered. ^1^ Phytozome gene identifier of Selaginella moellendorffii.

The identified LHT proteins are grouped in three main clusters (Figure 1). Cluster 1 includes LHT proteins of seed plants, cluster 2 contains some non-seed vascular plant and all non-vascular LHT proteins, and cluster 3 contains seed plant and the remainder of the non-seed vascular plant Selaginella proteins. Cluster 1 contains 8 of the 10 Arabidopsis, 6 of the 7 Medicago, 4 of the 6 rice, 5 of the 6 tomato and all of the sorghum LHTs (Figure 1). Thus, cluster 1 was found to contain the largest number of *LHT* genes (Figure 1). In addition, the simultaneous presence of monocots and eudicots in most clades of the cluster 1. These results indicated that the formation of the LHT family occurred before the split of dicots and monocots and that cluster 1 is only present in flowering plant species. Cluster 2 includes all Physcomitrella *LHT* genes and 6 out of 8 Selaginella *LHT* genes, but no genes from seed plants (Figure 1). It appears that the *LHT* genes in cluster 2 may have evolved independently in Physcomitrella and Selaginella. Meanwhile, the transport functions of *LHT* genes from cluster 2 may not be required in higher plants, though they are required in early land plants. Cluster 3 contains 2 of the 10 Arabidopsis, 1 of the 7 Medicago, 2 of the 6 rice, 1 of the 6 tomato and 2 of the 8 Selaginella *LHT* genes (Figure 1). This indicates that the *LHT* genes in cluster 3 have other functions which are required by both non-seed vascular plants and seed plants. 

### 2.3. OsLHT Gene Structures and Protein Structures

**Figure 2:**
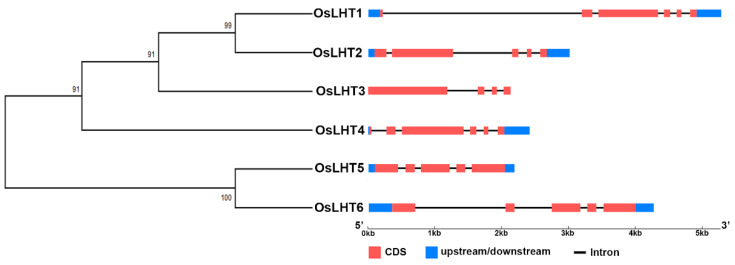
Exon-intron organization and phylogenetic analysis of Os*LHT* genes. The unrooted phylogenetic tree construction of the six rice LHT proteins was performed according to the neighbor-joining method in MEGA X software. Red boxes and black lines, respectively, stand for exons and introns. Blue boxes represent untranslated regions (UTRs). The scale at the bottom of the figure can be used to estimate the size of each Os*LHT* gene.

To investigate the structural diversity of the *OsLHT* genes, an unrooted phylogenetic tree was constructed by aligning the full-length sequences of the six OsLHT proteins. The members of the OsLHT family can be classified into two clusters containing four and two members, respectively (Figure 2). We compared the transcript and genomic sequences of each *OsLHT* gene to examine the intron/exon organization using the GSDS website (http://gsds.cbi.pku.edu.cn/) (accessed on 5 August 2022). The results showed that there are between three and five introns in the coding sequences of the six *OsLHT* genes. Members of the same subfamily have similar intron/exon structures. Of note, only the *OsLHT3* gene is not upstream and downstream. 

**Figure 3:**
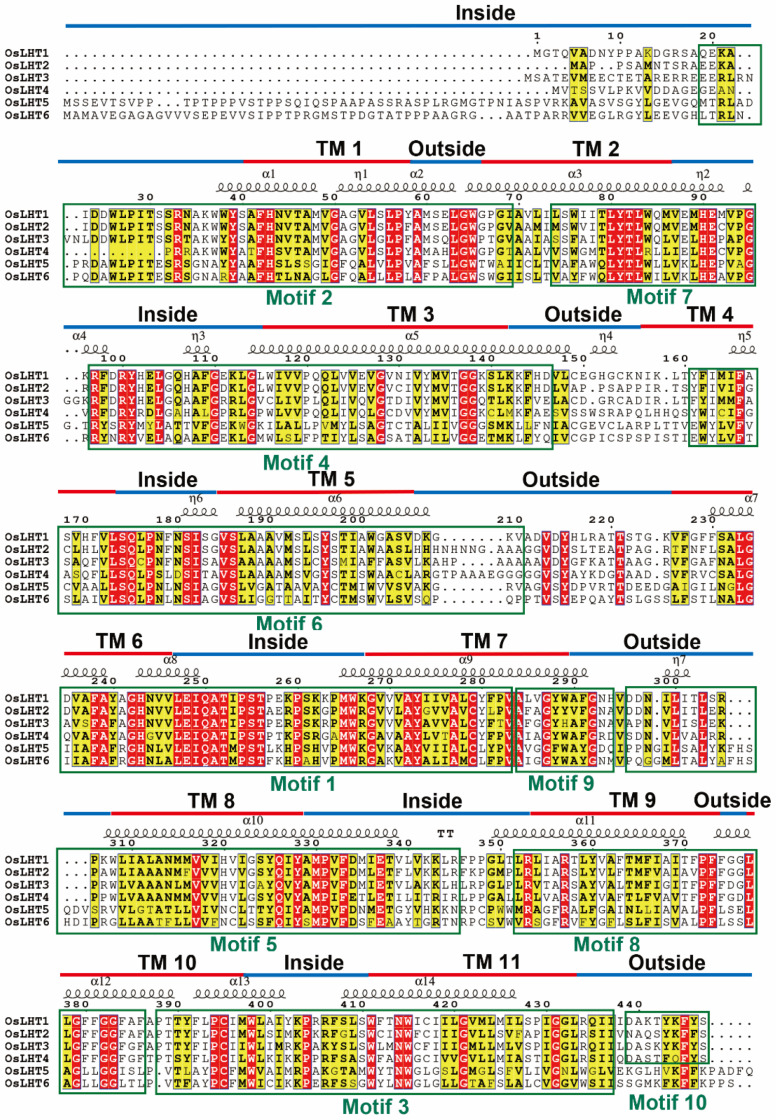
The multiple-sequence alignments for OsLHT proteins in rice. The secondary structures, conserved motifs and TM regions are marked by symbols, boxes and lines, respectively.

We identified 10 distinct motifs using the MEME website to investigate the diversification of the OsLHT proteins (Figure 3). The lengths and amino acid sequences of all 10 putative motifs are listed in Figure S2. The potential functions of each motif were determined by SMART and Pfam, revealing that seven motifs (2–7 and 9) encode Aa-trans domains, while the remaining three motifs do not. Motifs 1–9 were widely distributed in all the OsLHT proteins, while motif 10 was only found in the OsLHT1-4 protein. The results indicated that the most conserved motifs found in the OsLHT proteins were highly compatible with their evolutionary relationships and that the cluster had similar conserved motifs in terms of both numbers and varieties.

**Figure 4:**
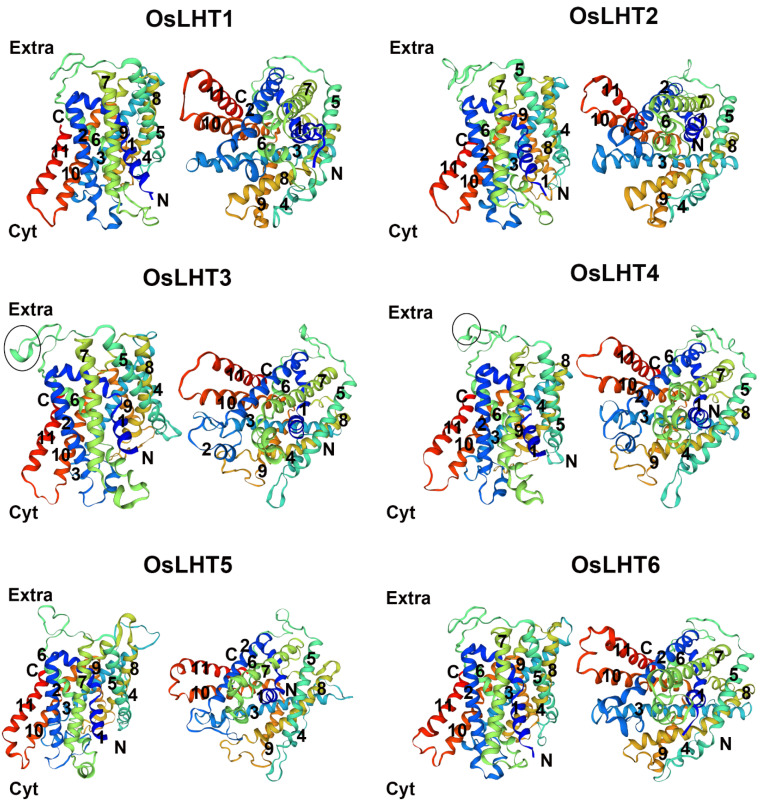
Three-dimensional modeling of OsLHT proteins. Side view (**left**) and top view (**right**) of the model created using SwissModel. The partial a-helix in the extracellular portion between the fifth transmembrane region and the sixth transmembrane region is marked by a circle.

Previous studies have reported that the members of the *LHT* family can transport amino acids in Arabidopsis and rice [16,18]. To verify the transport function of *LHT* genes, the membrane-protein topology prediction tool DeepTMHMM was used to predict the putative TM regions; each OsLHT protein had 11 helical TMs. To further examine the relationship between the conserved motifs and the TM areas of OsLHT proteins, multiple-sequence alignment was performed and highlighted the secondary structures, conserved motifs and TM regions, as shown in Figure 3. The TM regions and conserved motifs were found to be highly correlated. Motifs 4 and 5 included the complete TM 3 and TM 8 regions, while the majority of the TM 2 and TM 7 areas were found in motifs 7 and 9. The tenth TM area was the location of motif 3, which continued into the subsequent sequences after the eleventh TM region. In addition, motif 1 contained the most regions of TM 6 and TM 7, while motifs 2, 6 and 8, respectively, contained the complete TMs 1, 5 and 9 and most regions of TMs 2, 4 and 10. The OsLHT proteins had a total of 14 α-helices and 7 η-helices, and only α2, α4, η2, η3, η4, η6 and η7 were found in the non-transmembrane regions, while the remaining secondary structures were found in the transmembrane areas to maintain the transport function of the LHT proteins (Figure 3). The structures of the OsLHT proteins were predicted using SwissModel servers (Figure 4). It became apparent that all predicted OsLHT proteins had 11 helical TMs and displayed complex 3D structures, including multiple secondary structures (Figure 4). In addition, in contrast to OsLHT1, 2, 5 and 6, OsLHT3 and OsLHT4 had a partial a-helix in the extracellular portion between the fifth transmembrane region and the sixth transmembrane region (Figure 4).

### 2.4. The Subcellular Localization of OsLHTs

**Figure 5:**
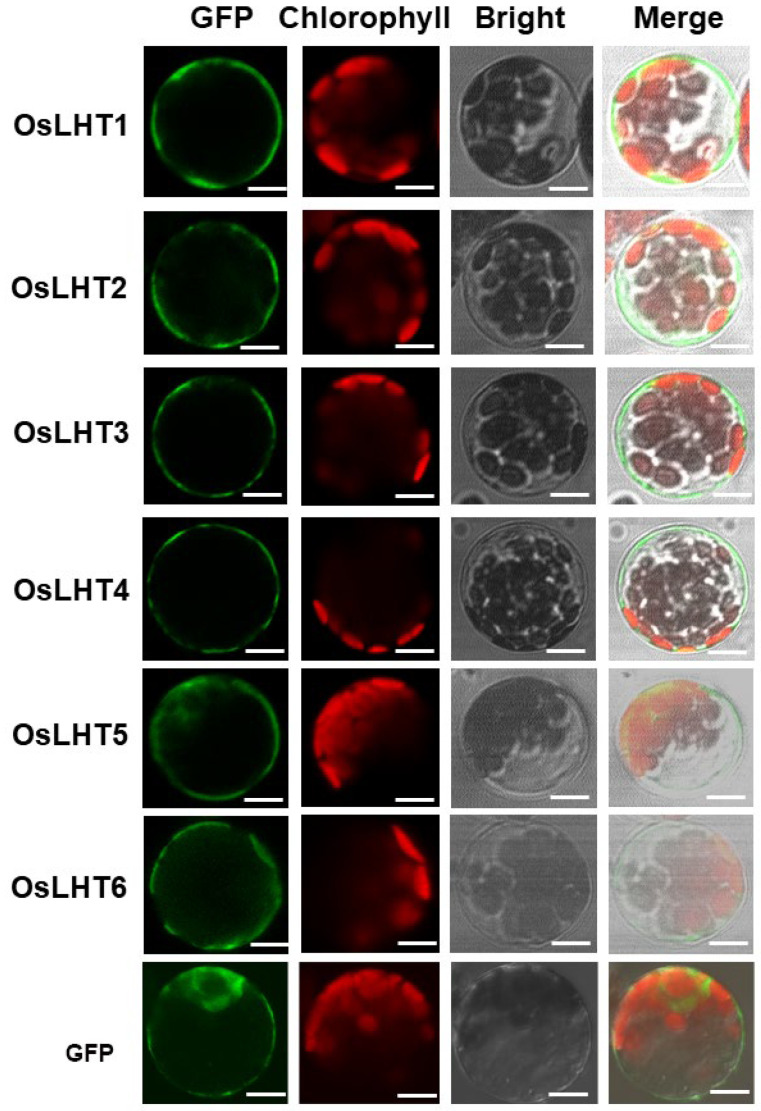
Subcellular localization of OsLHTs in Arabidopsis protoplasts. Abbreviations: GFP, GFP fluorescence image; Chlorophyll, chlorophyll autofluorescence; Bright, bright-field image; Merged, merged bright-field, GFP fluorescence and chloroplast fluorescence images. Free GFP was used as a positive control. Bars = 5 μm.

To understand the basal function of OsLHT proteins, we first predicted their possible subcellular localizations using the WoLF PSORT website. The prediction results indicated that most OsLHTs are mainly localized in the cell plasma membrane (Table 1). Next, the fusion proteins of OsLHTs and GFP under the CMV 35S promoter were constructed and transiently expressed in Arabidopsis protoplasts. The GFP fluorescence signal was observed after 12–18 h incubation. The protoplasts expressing GFP alone acted as a positive control. The results showed that OsLHT1-4 were localized in the plasma membrane, while OsLHT5 and OsLHT6 were localized in the plasma membrane and cytosol (Figure 5). 

### 2.5. The Transport Functions of OsLHTs

**Figure 6:**
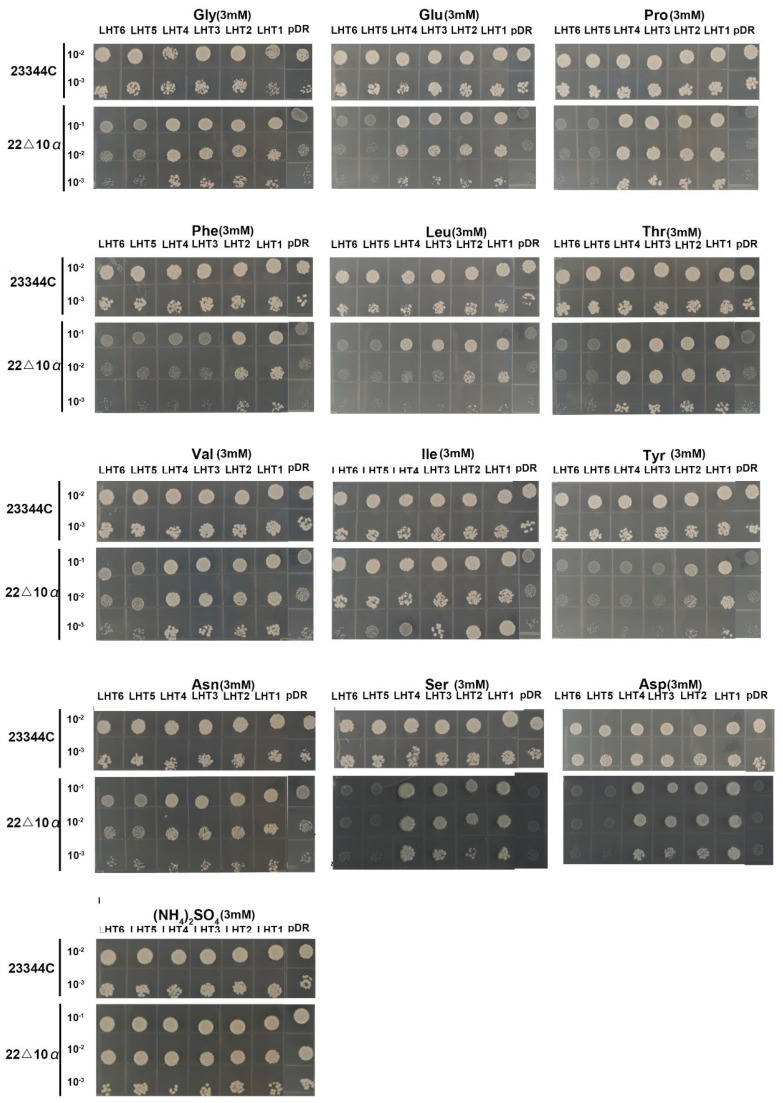
Yeast function complementation experiment. The amino acids acted as the only nitrogen source in the media. The pDR195−OsLHTs and the empty vector pDR195 were transformed into the wide-type strain 23344c and the mutant strain *22Δ10α*, respectively. Yeast clones were cultured overnight in a selective medium to OD = 1, then diluted to OD = 0.1, 0.01 and 0.001. Drops of 7 μL were aligned on minimal media with the amino acids as the sole nitrogen source. Images were taken after 7 days of growth at 30 °C.

To investigate the transport functionalities of *OsLHTs*, yeast function complementation experiments were conducted using the yeast mutant *22Δ10a*, which has an amino acid transport defect. *22Δ10a*, lacking ten plasma membrane amino acid transporters, is unable to grow on media containing proteinogenic amino acids or c-aminobutyric acid (GABA) as the sole N source [26]. We cloned the six *OsLHT* genes into a yeast expression vector pDR195 and transformed it into the wild-type strain 23344c and the mutant strain *22Δ10a*. Next, yeast cells expressing the six *OsLHT* genes and the empty vector pDR195 were grown on solid media containing acidic, basic and neutral amino acids at 3 mM concentrations. The results indicated that the six members of the *OsLHT* gene family have different amino acid selectivities. *OsLHT1*-expressing yeast was able to grow on all tested amino acid media, including the acidic amino acid and most of the neutral amino acid media (Figure 6). The broad amino acid selectivity of *OsLHT1* is also consistent with the substrate analysis in *Xenopus* oocytes by Wang et al. [18]. Similar to *OsLHT1*, *OsLHT2* also showed broad amino acid selectivity (Figure 6). In contrast to *OsLHT1*, yeast cells expressing *OsLHT3* and *OsLHT4* genes displayed similar growth patterns to the empty vector pDR195 on the media with Phenylalanine and tyrosine (Figure 6). By contrast, yeast cells expressing *OsLHT5* and *OsLHT6* genes were not able to grow on the media with the tested amino acids as the only N source (Figure 6).

To further investigate the effect of the different amino acids as the sole nitrogen sources on *OsLHT* genes expression, 7 days after germination, rice seedlings were nitrogen-starved for 24 h and then provided with ammonium nitrate or various amino acids as a sole N source. After 24 h of incubation, *OsLHT1* transcript levels were obviously higher in plants incubated with various amino acids, including Ile, Phe, Asp, Ala, Gln, Pro, Val, Thr and Gly, than those incubated in the ammonium nitrate (Figure S3). The *OsLHT4* gene exhibited minor upregulation in the expression of several amino acids (Ile, Phe, Ala, Asn, Glu, Val and Thr) as compared with the ammonium nitrate condition (Figure S3). On the contrary, the expression levels of *OsLHT5* and *OsLHT6* with respect to the detected amino acids did not increase compared with the ammonium nitrate condition (Figure S3). We did not analyze *OsLHT2* and *OsLHT3* due to their expression levels in roots 7 days after germination being very low. These results indicated that *OsLHT5* and *OsLHT6* may transport other substrates than amino acids.

### 2.6. Analysis of Promoter Cis-Elements in the OsLHT Gene Family

**Figure 7:**
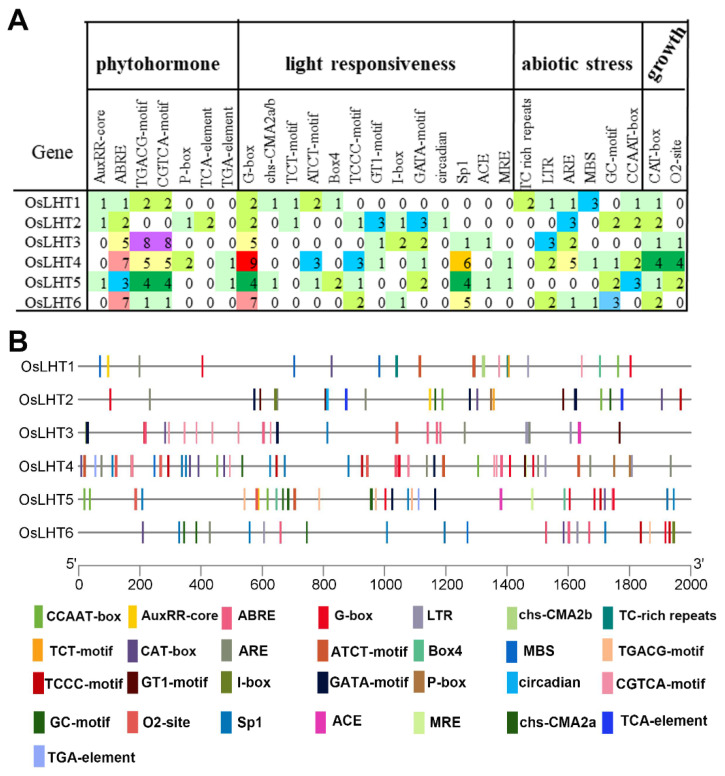
Cis-acting elements identified in the promoter regions of the six OsLHT genes in rice. (**A**) Information on species and localizations. (**B**) Information on quantities.

To further elucidate the regulatory mechanisms of the *OsLHT* genes in rice, we extracted and analyzed the upstream 2000 bp sequences of the ATG initiation codons of the six *OsLHT* genes using PlantCARE for cis-element analysis. The results showed that the detected cis-elements could be divided into four categories: phytohormones, light responsiveness, abiotic stress and plant growth (Figure 7, Table S2). The phytohormone-responsive cis-elements included the AuxRR-core/TGA-element (auxin response), ABRE (abscisic acid response), the P-box (gibberellin response), the TGACG-motif/CGTCA-motif (MeJA response) and the TCA-Element (salicylic acid response). The light-responsive cis-elements included the G-box, chs-CMA2a/b, TCT-motif, ATCT-motif, Box4, TCCC-motif, GT1-motif, I-box, GATA-motif, circadian, Sp1, ACE and MRE. The stress-related elements included the TC-rich repeats (defense and stress response), LTR (low temperature), CCAAT-box (MYBHv1 binding site), ARE/GC motifs (anoxic/anaerobic response) and MBS (drought response). The plant growth categories included CAT-box (meristem expression) and O2-site (zein metabolism). The types and locations of these elements are shown in Figure 7A, and the number of elements is displayed in Figure 7B. The light-responsive elements were the most abundant cis-elements in the *OsLHT* genes. Each gene had more than four light-responsive elements. The elements related to abiotic stress were also distributed in *OsLHT* genes, especially in LTR and ARE/GC motifs. We also found that many phytohormone-responsive cis-elements related to abscisic acid, auxin and methyl jasmonate (MeJA) were widely distributed in *OsLHT* genes, suggesting that these *OsLHT* genes may be involved in hormone signaling and stress response. 

### 2.7. Expression Profiling of OsLHT Genes

**Figure 8:**
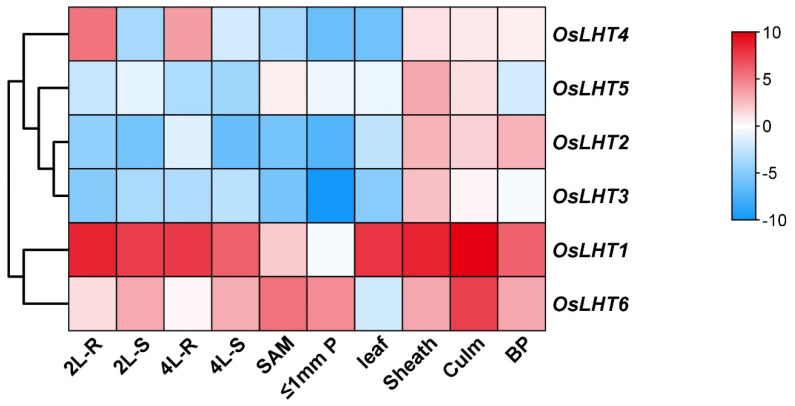
Heat map of the quantitative real-time PCR analysis results for the *OsLHT* genes at various developmental stages and in various organs, with three biological and technical replicates. The scale above displays the relative signal intensity values. Abbreviations: 4L−R, roots of 4−leaf stage seedlings; 4L−S, 4−leaf stage seedlings; 2L−R, roots of 2−leaf stage seedlings; 2L−S, 2−leaf stage seedlings; 10L−SA, shoot apex of 10−leaf stage seedling; BP, booting panicle; ≤1P, developing panicle with a length of ≤1 cm.

As we know, the comprehensive analysis of gene expression patterns in different tissues will help us to further understand the functions of *LHT* genes in rice. The expression patterns at various developmental stages and in various organs were examined to assess the transcription of *OsLHT* genes throughout the entire rice life cycle (Figure 8 and Figure S4). The results demonstrated that *OsLHT* genes displayed abundant expression profiles in various tissues. The expression profiles of the *OsLHT1* genes exhibited strong constitutive expressions in all tested tissues. *OsLHT2* and *OsLHT3* were specifically expressed in the reproductive organs of the panicles, leaf sheaths and culms. *OsLHT4* displayed higher expression in roots in the vegetative period and in the leaf sheaths and culms in the reproductive period. *OsLHT5* was predominantly expressed in leaf sheaths. *OsLHT6* displayed high expression in all tested tissues except the leaf, its highest expression levels being in the culm. *OsLHT* genes displayed a variety of expression patterns, including tissue-specific expression and constitutive expression. 

**Figure 9:**
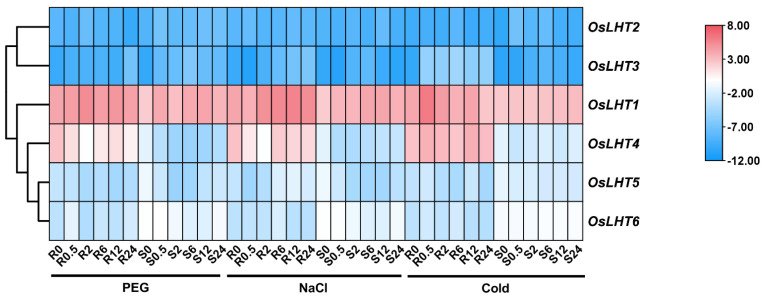
Heat map of the quantitative real−time PCR analysis results of the *OsLHT* genes in roots and shoots under cold, NaCl and PEG treatments, with three biological and technical replicates. The scale above displays the relative signal intensity values.

For the abiotic stress treatment (Figure 9 and Figure S5), the *OsLHT* genes showed different response patterns under PEG, NaCl and cold stress. Following PEG treatment, the transcript levels of *OsLHT1* and *OsLHT2* were slightly upregulated during early treatment, followed by a decrease in the roots and shoots. The expression of *OsLHT3* peaked at 24 h in the roots, while it was highly expressed at 6 h in the shoots. For *OsLHT4* and *OsLHT5*, the expression levels gradually decreased from the initial time point to 2 h in the roots and 6 h in the shoots. The expression of *OsLHT6* was initially upregulated to high levels at 0.5 h and then downregulated in the roots and was gradually downregulated in the shoots. Following NaCl treatment, three genes (*OsLHT1*, *OsLHT2* and *OsLHT6*) showed slight changes in expression levels in both the roots and shoots. *OsLHT3* reached its peak during the initial (0.5 h) treatment, followed by a decrease in expression during the subsequent treatment for the shoots. By contrast, the expression of *OsLHT3* was the lowest at 2 h, with a gradual increase at all later time points for the roots. The expressions of *OsLHT4* and *OsLHT5* were strongly downregulated at 0.5 h in both roots and shoots. Under cold stress, there were three genes (*OsLHT4*, *OsLHT5* and *OsLHT6*) that exhibited minor changes in expression levels in both roots and shoots at all time points. For the roots, *OsLHT1* and *OsLHT3* were obviously upregulated; their expression levels peaked at 0.5 h and 6 h. For the shoots, only the expression of *OsLHT2* exhibited obvious upregulation, at 0.5 h.

**Figure 10:**
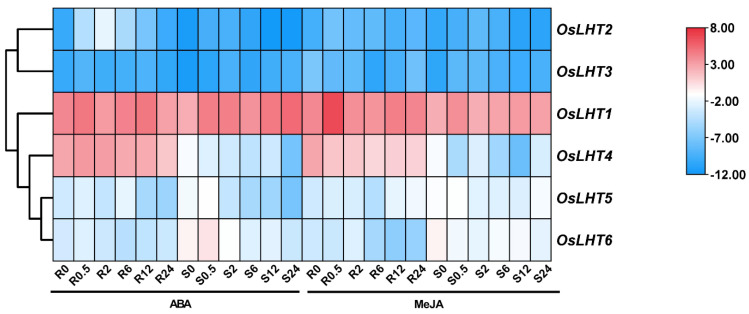
Heat map of the quantitative real−time PCR analysis results of the *OsLHT* genes in roots and shoots under MeJA and ABA treatments, with three biological and technical replicates. The scale above displays the relative signal intensity values.

From the heat map of the qRT-PCR analysis results for the *OsLHT* genes (Figure 10 and Figure S6), a few genes were found to be ABA- and MeJA-responsive. Following ABA treatment, all the genes except *OsLHT2* showed slight changes in their expression levels in both roots and shoots. *OsLHT2* was upregulated during the initial phase of the treatment, peaking at 2 h, but was downregulated at later time points for the roots. Under MeJA treatment, *OsLHT5* and *OsLHT6* showed slight changes in expression levels in both roots and shoots. The expression of *OsLHT4* was downregulated at 0.5 h in both roots and shoots. For the roots, *OsLHT1*, *OsLHT2* and *OsLHT3* were obviously upregulated; their expression levels peaked at 0.5 h. For the shoots, *OsLHT2* and *OsLHT3* were upregulated at 2 h and 0.5 h. These results suggest that some *OsLHT* genes might play important roles in responses to cold, PEG, ABA, NaCl and MeJA treatments.

## 3. Discussion

Amino acid transporters are some of the main determinants of N distribution and play a pivotal role in the process of plant development and growth. AATs are widely distributed in many higher plants and have been thoroughly systematically identified and characterized in many plants, such as Tartary buckwheat (104) [27], Arabidopsis (65) [4], soybean (189) [6], potato (72) [8], rice (85) [5], common wheat (297) [9], etc. Of eight subfamilies of AATs, the AAP family has received the most focus in plants. Compared with the AAP family, further functional studies on the LHT family are required. Thus, in this study, we focused on rice crops, and performed a systematic identification and comprehensive characterization of the *LHT* gene family. Zhao et al. reported the genomic frameworks, classifications, duplication manners, conserved motifs and expression profiles of all 85 OsAAT members, including the subfamilies of AAPs, LHTs, ProTs, GATs, AUXs, ANTs, ATLs, CATs, ACTs and PHSs [5]. However, in our study, we mainly focused on the LHT subfamily of amino acid transporters. In addition to identifying the chromosomal locations, physicochemical properties, duplication events, phylogenetic relationships, cis-acting elements of promoters, gene structures and protein three-dimensional structures of the *OsLHT* genes, we also analyzed the transport functions of these genes through various experiments, such as subcellular localizations, transport substrate assays and analyses of gene expression patterns in various amino acid conditions. Heterologous yeast complementation experiments were performed to determine whether OsLHT proteins are functional transporters and their substrate specificities. Transient expression in Arabidopsis protoplasts suggested that most of the OsLHT proteins were localized to the plasma membrane, which is consistent with their functions as amino acid transporters. Therefore, our results can help to understand the substrate selectivity of *OsLHT* genes for various amino acids and thus provide theoretical guidance for studying the role of *OsLHT* genes in the uptake and partitioning of amino acids for plant growth and development.

The numbers of members of the *LHT* gene family in different plants varies, from 6 in rice to 24 in soybean and wheat. The number of *LHT* genes in rice was the lowest compared with other plants. Furthermore, *OsLHT* genes were classified into three clusters according to phylogenetic analysis of LHT proteins from *Physcomitrella patens, Selaginella moellendorffii, Arabidopsis thaliana, Oryza sativa, Medicago truncatula, Solanum lycopersicum* and *Sorghum bicolor* (Figure 1). The *LHT* gene family evolved before the differentiation of dicots and monocots, as evidenced by the clustering of LHT genes in the same clade in both dicots and monocots. The phylogenetic analysis showed the evolutionary relationships between the rice LHT proteins, and also indicated that cluster I contained the largest number of *LHT* gene members and that all the members came from seed plants. For the eight Arabidopsis genes, three pairs (*AtLHT2*/*AtLHT5*, *AtLHT1*/*AtLHT10* and *AtLHT3*/*AtLHT6*) were generated by a duplication event. By contrast, no duplicated gene pairs of LHT were found in rice (Figure 1). This suggested that the replication of *LHT* genes in monocots is not as extensive as in eudicots. Research on the function of plant *LHTs* was mainly performed in Arabidopsis; some studies have shown some that Arabidopsis *LHT* transporters in cluster 1 are expressed in male and female floral tissues, indicating a role in reproduction; for example, the fact that *LHT2* and *LHT6* were found to be expressed in tapetal cells strongly suggests that amino acids are transported in the tapetum to maintain pollen development [28]. *LHT6* is expressed in abundance in anther tissues, where it may play a role in the supply of amino acids for growth. *LHT5* is expressed in pollen tubes and may be involved in the acquisition of amino acids necessary for tube elongation. *LHT5* and *LHT6* are expressed in transmission tissues [29]. In our study, four rice *LHT* transporters in cluster 1 were found to be mainly expressed in the reproductive organs of the panicles, leaf sheaths and culms. *OsLHT1* genes exhibited strong constitutive expression in various tissues (Figure 8 and Figure S4). *OsLHT2* and *OsLHT3* were mainly expressed specifically in the reproductive organs of the panicles, leaf sheaths and culms (Figure 8 and Figure S4). *OsLHT4* displayed higher expression in roots in the vegetative period and in the leaf sheaths and culms in the reproductive period (Figure 8 and Figure S4). This suggested that the *LHT* genes might be crucial for effective sexual plant reproduction. Additionally, this is consistent with a study on Arabidopsis [29]. Although the expression pattern of *LHT* indicates that it may participate in reproductive development, there is no report on its function in reproduction. Moreover, *OsLHT1* and *OsLHT4* seem to have additional functions, as they are expressed in other organs, such as roots and leaves in the vegetative period. For example, some studies have shown that *OsLHT1* is a key transporter in root uptake and root-to-shoot amino acid allocation. It also mediates the translocation of amino acids from vegetative to reproductive organs for nutrition quality and seed yield [19,20]. Cluster 2 includes total Physcomitrella *LHT* genes and parts of Selaginella *LHT* genes but none from seed plants (Figure 1). It appears that the transport functions of *LHT* genes from cluster 2 may not be required in higher plants, though they are required in early land plants. Cluster 3 contains both non-seed vascular plants and seed plants (Figure 1), indicating that the *LHT* genes from cluster 3 have other functions which are required by both non-seed vascular plants and seed plants. Arabidopsis *AtLHT4* and *AtLHT7* are not only involved in anther and pollen development; they are also expressed in roots and stems [30]. Similarly, *OsLHT5* was found to be predominantly expressed in leaf sheaths (Figure 8 and Figure S4). *OsLHT6* displayed high expression levels in all tested tissues except leaf tissues, with its highest expression in the culm (Figure 8 and Figure S4). *OsLHT5* and *OsLHT6* within cluster 3 shared similarities in motif distribution (Figure 3), gene structure (Figure 2) and transport substrate (Figure 6), implying the reliability of the subfamily classification. The diverse functions of *LHT* genes still need further research.

As a class of amino acid transport proteins, the transport substrate determines the function of the gene to a certain extent. Several *LHT* members, including *AtLHT1* [16], *AtLHT6* [25] and *OsLHT1* [20], have been reported to take up amino acids from external media. In fact, *OsLHT1*-related uptake seems to contribute to about 45% of total aspartate uptake [20]. This suggests that *LHT* genes plays an important role in the uptake of amino acids by roots. Although the underlying molecular mechanisms of amino acid uptake are unknown, the identification of transport substrates can help us better understand the functions of genes. In Arabidopsis, *AtLHT2* transports neutral amino acids with high affinity [28]. *AtLHT6* absorbs acidic and some neutral amino acids, but not basic amino acids [25]. In rice, the substrate specificities of six *LHT* members have not been determined until now, except that of *OsLHT1*. Similar to *AtLHT2* and *AtLHT6*, *OsLHT1* was confirmed to transport a wide range of amino acids, including basic, neutral and acidic amino acids, and displayed a preference for neutral and acidic amino acids in yeast and Xenopus oocytes [18]. Our yeast complementation study produced similar results (Figure 6). Regarding the other members of the *OsLHT* gene family, *OsLHT2* also showed broad amino acid selectivity. OsLHT3 and OsLHT4 were able to transport all of the test amino acids except phenylalanine and tyrosine. By contrast, OsLHT5 and OsLHT6 were not able to transport all the test amino acids (Figure 6). Meanwhile, the expression levels of *OsLHT5* and *OsLHT6* in the different amino acids as sole N sources did not increase compared with the ammonium nitrate condition (Figure S3). Previous studies have reported that, besides amino acids, other substrates, including ACC [21,22], indole-3-acetic acid (IAA) [31], choline [32], glycine betaine [32,33] and amino acid-based pesticides [24,34], can also be transported by some amino acid transporters. ACC, another non-proteinogenic a-amino acid, has been shown to be transported by AtLHT1 and AtLHT2 [21,22]. Recently, *AtLHT1* has been found to facilitate the movement of pesticide–amino acid conjugates within plants [24]. Thus, we speculated that OsLHT5 and OsLHT6 may transport other substrates besides amino acids. The diversity of substrates suggests that *OsLHT* genes may be involved in various physiological progress in plants. Although in this study we performed a preliminary analysis of the substrates of the six *LHT* genes in rice by yeast complementation experiments, further verifications and determinations of the affinities of the substrates via Xenopus oocyte injection experiments are required. To better study the gene functions, the uptake and in vivo transport of ^15^N-labeled amino acids in the *lht* knockout mutant could be examined.

As we know, drought, salt and cold are the most severe abiotic stresses in agricultural production. The accumulation of proline plays an important role in protecting plants from abiotic stresses [35]. Proline may accumulate in plants due to improved synthesis and reduced degradation as well as proline transport within plants [35]. The cis-elements in promoters will regulate gene expression when they are stimulated by various environmental conditions. We identified cis-elements in the promoters of *OsLHT* genes, which were involved in light responsiveness, hormonal responses and environment responses (Figure 7). These identifications indicated that *OsLHT* genes might be involved in the regulation of abiotic stress. Therefore, we used qRT-PCR and searched the TENOR (https://tenor.dna.affrc.go.jp/) (accessed on 9 November 2022) and RiceXPro (https://ricexpro.dna.affrc.go.jp/) (accessed on 9 November 2022) databases to examine the expression profiles of *OsLHT* family members under cold, NaCl and PEG stresses (Figure 9 and Figure S5). The results showed that *OsLHT1* and *OsLHT3* were upregulated in roots in response to the PEG, NaCl and cold treatments and that *OsLHT6* was upregulated in roots in response to the PEG treatment (Figure 9 and Figure S5). OsLHT1 has been reported to transport proline [18]. Our study also demonstrated that OsLHT1 and OsLHT3 can transport proline, but that OsLHT6 cannot transport proline. Therefore, we speculated that increased expression of *OsLHT1* enhances the external uptake of proline by roots in response to various stresses. In addition, we predicted the miRNAs that can interact with *OsLHT1* using PmiREN (https://pmiren.com/) (accessed on 9 November 2022) and found that *OsLHT1* may be the target gene of miR396 (Figure S7). miR396 has also been reported to regulate growth, development and abiotic stress tolerance in Arabidopsis [36]. Therefore, *OsLHT1* may also potentially respond to adversity by interacting with miR396. OsLHT3, because it is specifically expressed in reproductive organs, transports more amino acids to developing reproductive organs through increased expression in response to stress. Previous studies have revealed that over-expression of the GABA transporter *PeuGAT3* enhances abiotic stress tolerance in Arabidopsis [37]. GABA acts as an endogenous signaling molecule to regulate plant stress responses [38]. Although OsLHT6 cannot transport proline, it may transport other stress-induced compounds (e.g., GABA) in response to stress. On the contrary, *OsLHT4* was downregulated in roots and shoots in response to the PEG and NaCl treatments (Figure 9 and Figure S5), while *OsLHT5* was downregulated in shoots in response to the PEG and NaCl treatments (Figure 9 and Figure S5). This suggested that *OsLHT4* and *OsLHT5* may be negative regulators of abiotic stress. Thus, it is possible that these *OsLHT* genes respond to abiotic stress and are key genes involved in stress resistance in rice. The newly discovered data generated in this study may provide a foundation for the further functional characterization of *OsLHT* genes.

## 4. Materials and Methods

### 4.1. Identification of LHT Genes in Rice 

In this study, we performed name searches with keywords (e.g., Lysine/Histidine transporter) to obtain annotated candidates of *AtLHT* and *OsLHT* family members in the Arabidopsis Information Resource (TAIR, http://www.arabidopsis.org/) (accessed on 25 June 2022) and RAP-DB (http://rapdb.dna.affrc.go.jp/) (accessed on 20 June 2022), respectively. As 10 *AtLHT* (*AtLHT1–10*) and 6 *OsLHT* (*OsLHT1-6*) transporters have been previously identified, we used the gene nomenclature for Arabidopsis LHTs proposed by Rentsch et al. [4] and that for rice LHTs proposed by Zhao et al. [5]. LHT sequences were selected from *Physcomitrella patens*, *Selaginella moellendorffii*, *Medicago truncatula*, *Solanum lycopersicum* and *Sorghum bicolor* protein sequences via BLAST searches for Arabidopsis LHTs on the Phytozome v13.1 website (http://www.phytozome.net/) (accessed on 30 June 2022). Information on rice *LHT* genes, including open reading frame (ORF) lengths, locations and numbers of amino acids were obtained from RAP-DB. The physicochemical parameters of each OsLHT protein were found using ExPASy (http://web.expasy.org/protparam/) (accessed on 20 August 2022). The subcellular localization of the OsLHT proteins was predicted using WoLF PSORT (http://wolfpsort.org) (accessed on 9 September 2022) with the amino acid sequences. 

### 4.2. Chromosomal Localization and Gene Duplication

According to their positional data found in the Rice Genome Annotation Project (RGAP) database (http://rice.plantbiology.msu.edu/index.shtml) (accessed on 10 October 2022), the *OsLHT* gene chromosomal distributions were determined using MapInspect software. The *OsLHT* genes were created by segmental duplicating events if they were located on duplicated chromosomal blocks that were available at MSU-RGA with the maximum length distance permitted between collinear gene pairs of 500 kb. When genes in a 100 kb region were separated by five or fewer genes, tandem duplicates were identified.

### 4.3. Phylogenetic Tree Analysis

To investigate the evolutionary relationships between LHT genes in various plant species, the LHT protein sequences of Arabidopsis thaliana, Oryza sativa, Physcomitrella patens, Selaginella moellendorffii, Medicago truncatula, Solanum lycopersicum and Sorghum bicolor were used to construct an unrooted phylogenetic tree. Multiple-sequence alignment was performed using Clustal X (1.83) software, and the tree was constructed according to the maximum-likelihood (ML) method with the *p*-distance substitution model in MEGA X software. We used 1000 replicates in a bootstrap analysis to determine a support value for each branch.

### 4.4. Gene Structures, Conserved Motifs and Three-Dimensional Modeling

The Gene Structures Display Server2.0 (GSDS2.0) (http://gsds.cbi.pku.edu.cn/) (accessed on 15 October 2022) was used to examine gene structures (exons/introns). Multiple Expectation Maximization for Motif Elicitation (MEME) (http://meme.nbcr.net/meme/cgibin/meme.cgi) (accessed on 17 October 2022) was used to analyze the conserved motifs of LHT proteins. Motif widths were defined between 6 and 200 residues, and the maximum motif number was determined to be 10. The information on OsLHT protein secondary structures was obtained from the Protein Data Bank (PDB, https://www.rcsb.org/) (accessed on 17 October 2022). The putative transmembrane (TM) regions of OsLHT proteins were predicted using the DeepTMHMM Server (https://dtu.biolib.com/DeepTMHMM) (accessed on 20 October 2022) with default settings. Multiple-sequence alignment was carried out using Clustal X (version 1.83) and performed with ESPript 3.0 [39]. The conserved motifs and TM regions were highlighted on the multiple-sequence alignment. In addition, to further verify the TM regions of the OsLHT proteins, the amino acid sequences were submitted to the SwissModel server (https://swissmodel.expasy.org/) (accessed on 25 October 2022) to predict the three-dimensional structures.

### 4.5. Functional Characterization of OsLHT Using the 22Δ10a Yeast Strain 

The Saccharomyces cerevisiae mutant *22Δ10a* (MATα gap1-1 put4-1 uga4-1 can1::HisG lyp1alp1::HisG hip1::HisG dip5::HisG gnp1Δ agp1Δ ura3-1) was used for functional characterization of OsLHTs [26]. The coding sequences of OsLHTs were amplified by PCR and cloned into the pDR195 yeast expression vectors. The six constructed vectors were transformed into the *22Δ10a* strain and the wild-type (WT) yeast strain 23344c (MATα, ura3) using the PEG/LiAc method. At the same time, the empty vector pDR195 was transformed into the WT yeast strain 23344c (MATα, ura3) and the mutant strain *22Δ10a* strain as positive and negative controls. For the yeast complementation experiment, transformed yeast was grown on solid selective medium at 30 °C for 3 d, then yeast clones were resuspended overnight in a selective medium to OD = 1, washed twice with water and 10-fold serially diluted with water. Drops of 7 μL of each dilution were dropped onto solid YNB minimal medium without (NH_4_)_2_SO_4_, supplemented with 3 mM of different amino acids as the sole nitrogen source and 2% D-glucose as the C source. Observations were made after 7 d incubation at 30 °C and photographs were taken.

### 4.6. Plant Materials and Growth Conditions

In order to investigate the spatiotemporal expression patterns of the *OsLHT* genes, rice (Zhonghua 11) seedlings were grown under normal conditions during natural growing seasons (March to July) to investigate the spatiotemporal expression patterns of *OsLHT* genes. Two-week-old ZH11 seedlings were subjected to the following treatments for gene expression analysis: salinity (150 mM NaCl), cold (4 °C) and PEG (20% PEG6000). ABA (50 M) and MeJA (50 M) were introduced in the study on gene expression in response to the hormones. After treatment, samples of roots and shoots were taken at 0, 0.5, 2, 6 and 24 h. These samples were then promptly frozen in liquid nitrogen and kept at −80 °C. For the analysis of gene expression with amino acid treatment, one-week-old ZH11 seedlings were starved of nitrogen for 24 h and then supplied with ammonium nitrate or various amino acids as the sole N source for 24 h. All roots were collected and immediately frozen in liquid nitrogen and stored at −80 °C.

### 4.7. RNA Isolation and Quantitative Real-Time PCR

Total RNA was extracted from various rice samples in the different treatments based on the instructions from the manufacturer of RNAiso Plus (Takara, Kusatsu, Japan, code no. 9108). M-MLV Reverse Transcriptase (Promega, Madison, WI, USA, cat. no. M1701) was used for reverse transcription, with 2 μg of total RNA to cDNA. qRT-PCR was performed with SYBR Premix Ex Taq II (Takara, Kusatsu, Japan, RR820A), using a Roche Light Cycler 480 Real-Time PCR system. *eEF-1a* (LOC_Os03g08020) was used as an internal control. The 2^−ΔΔCT^ method was used to determine the relative expression levels of genes. This experiment was performed in triplicate. All primers used are listed in Table S1.

### 4.8. Subcellular Localization Assays

The open reading frames of *OsLHTs* without stop codons were inserted into pGL–GFP vectors, resulting in constructs containing the green fluorescent protein (GFP) fused at the C–terminus of the proteins. The constructs were transferred into Arabidopsis mesophyll protoplasts using PEG–mediated transformation [40]. Protoplasts harboring the empty pGL–GFP vector (35S:GFP) were used as a control. The protoplasts were incubated at 22 °C for 12–18 h in the dark, and the subcellular distribution of the GFP fusion protein was examined using a confocal laser scanning microscope. Excitation was achieved using an argon laser at 488 nm (GFP), and the emission of GFP was detected from 492 to 550 nm. The auto-fluorescence of chlorophyll was simultaneously detected between 650 and 730 nm. 

### 4.9. Analysis of Cis-Elements in OsLHT Promoter Regions

The upstream sequences of the *OsLHT* genes were extracted using NCBI. The obtained sequences were further delivered to the PlantCARE database for predicting and analyzing the cis-elements [41].

## Figures and Tables

**Table 1:** Six identified members of the *LHT* gene family in rice.

Locus ID	Gene Name	Chr ^a^	ORF Length (bp)	Number of Amino Acids	Molecular Weight (Mw/ Da)	Theoretical pI	FL-cDNAc	WoLF PSORT
LOC_Os08g03350	*OsLHT1*	8	1344	447	49,830.95	9.20	AK102015	PM
LOC_Os12g14100	*OsLHT2*	12	1341	446	48,941.59	8.87	AK070297	plas: 9.5, cyto_plas: 5.5, E.R.: 2, golg: 2
LOC_Os05g14820	*OsLHT3*	5	1371	456	49,939.82	9.13	EU956703	plas: 12, golg: 2
LOC_Os04g38860	*OsLHT4*	4	1335	444	47,795.99	9.09	AK243224	plas: 8, vacu: 3, cyto: 1, E.R.: 1
LOC_Os04g47420	*OsLHT5*	4	1539	512	55,030.86	9.13	AK060598	chlo: 12.5, chlo_mito: 7.5
LOC_Os12g30040	*OsLHT6*	12	1527	508	54,950.29	9.06	AK100852	plas: 6, chlo: 3, cyto: 2, nucl: 1, mito: 1

^a^ The chromosome on which the gene is located.

## Data Availability

Not applicable.

## Supplementary Materials

The following supporting information can be downloaded at: https://www.mdpi.com/article/10.3390/plants12040817/s1, Figure S1: Chromosomal distribution of *LHT* genes in rice. Six *OsLHT* genes were mapped to 4 of the 12 rice chromosomes. Chromosome numbers are located at the bottom of each vertical bar. Figure S2: Motif distribution in LHT proteins of rice and the conserved amino acid sequences. Motifs of the OsLHT proteins were identified by the online MEME program. Figure S3: The expression of *OsLHTs* under different amino acid treatments. Figure S4: Expression patterns of six *OsLHT* genes in various tissues, as revealed by qRT-PCR. Figure S5: Expression patterns of six *OsLHT* genes under PEG, Cold and NaCl stress, as revealed by qRT-PCR. Figure S6: Expression patterns of six *OsLHT* genes under MeJA and ABA stress, as revealed by qRT-PCR. Figure S7: The degradome evidence of interaction between *OsLHT1* and miR396 from PmiREN. Table S1: All primers used in this study. Table S2: The detailed functional annotations of cis-elements in the promoter regions of *OsLHTs.*
