# Supplementary material for: The LHT Gene Family in Rice: Molecular Characterization, Transport Functions and Expression Analysis
Source: Plants (Basel). 2023 Feb 12;12(4):817. doi: 10.3390/plants12040817 (PMC9958582; doi:10.3390/plants12040817)
Supplement: Supplementary file 1 [file plants-12-00817-s001.zip › plants-2056511-supplementary.pdf]

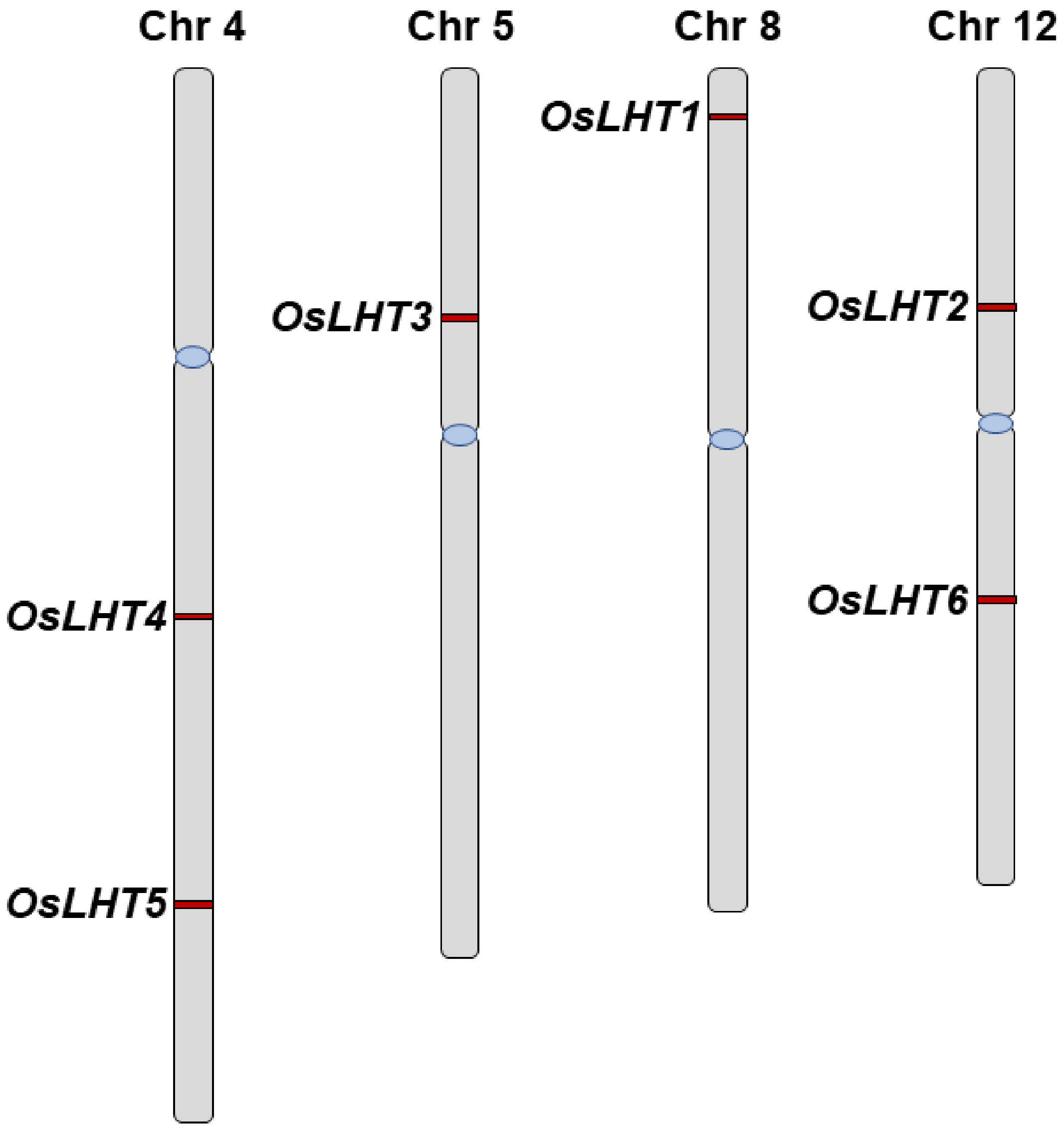

Figure S1. Chromosomal distribution of *LHT* genes in rice. 6 *OsLHT* genes are mapped to 4 of the 12 rice chromosomes. Chromosome numbers were located at the bottom of each vertical bar.

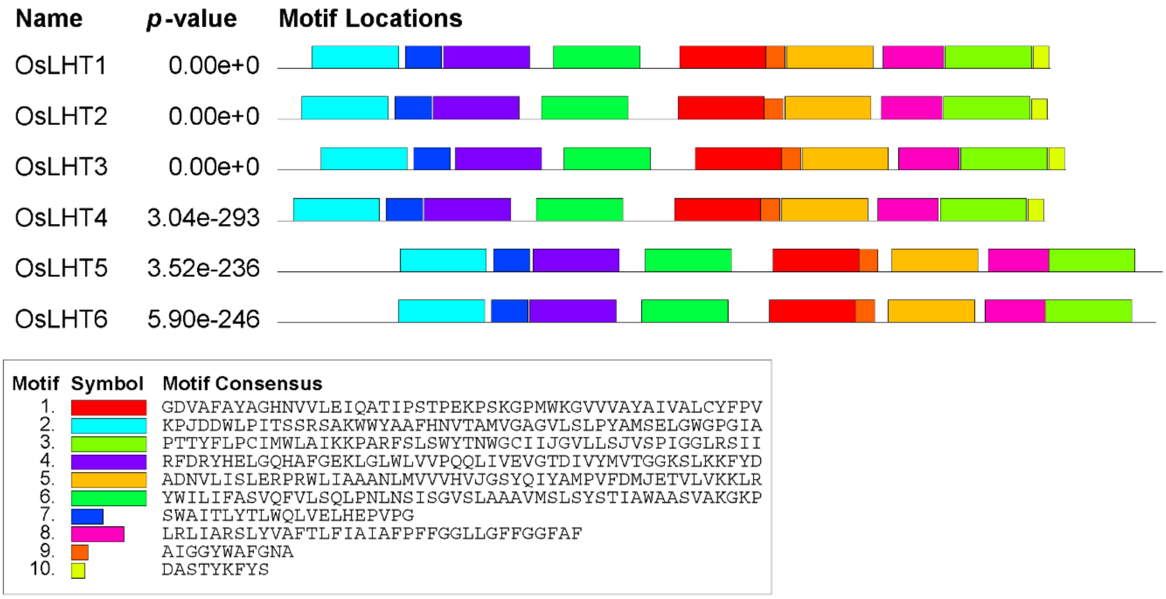

Figure S2. Motif distribution in LHT proteins of rice and the conserved amino acid sequences. Motifs of the OsLHT proteins were identified by the online MEME program.

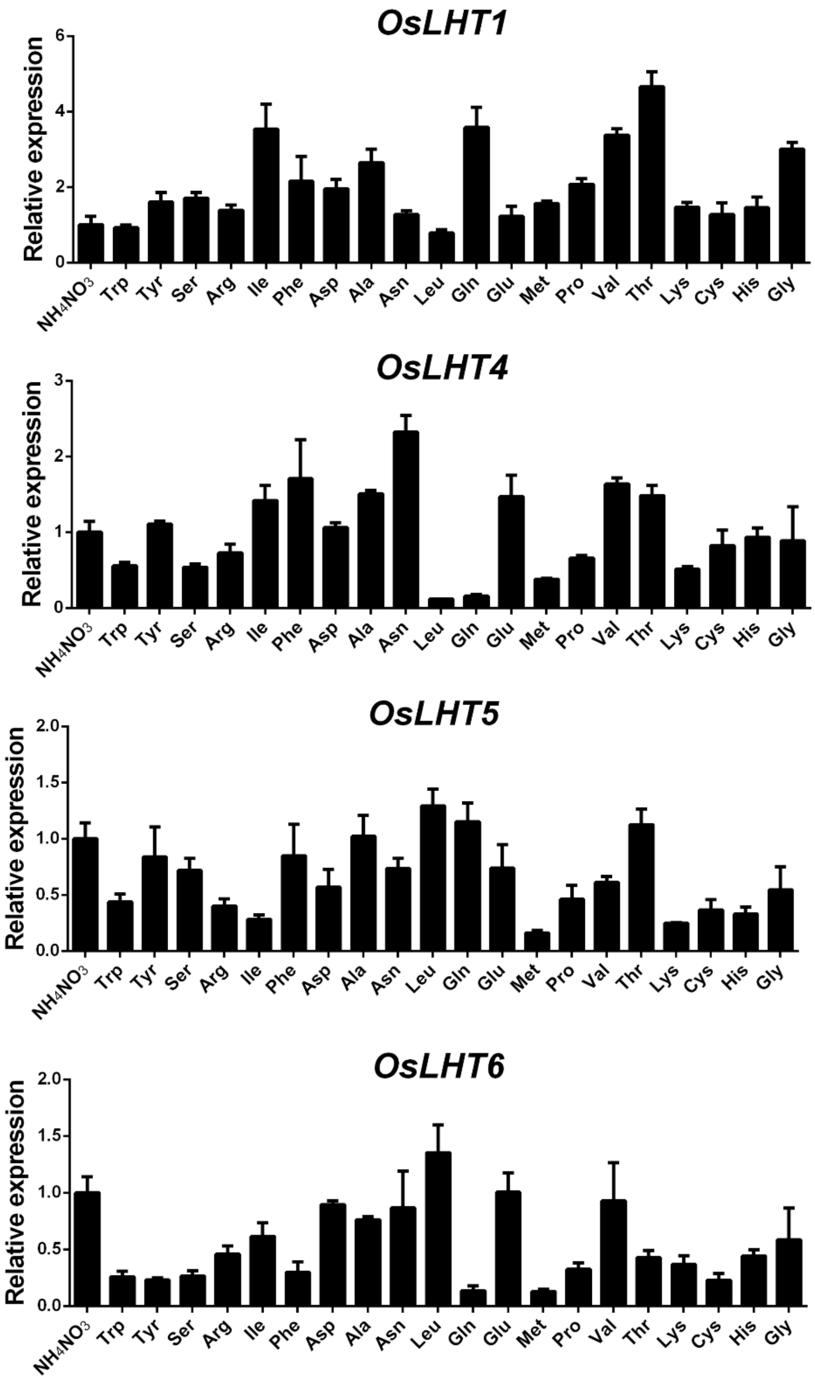

Figure S3. The expression of *OsLHTs* under different amino acids treatment.

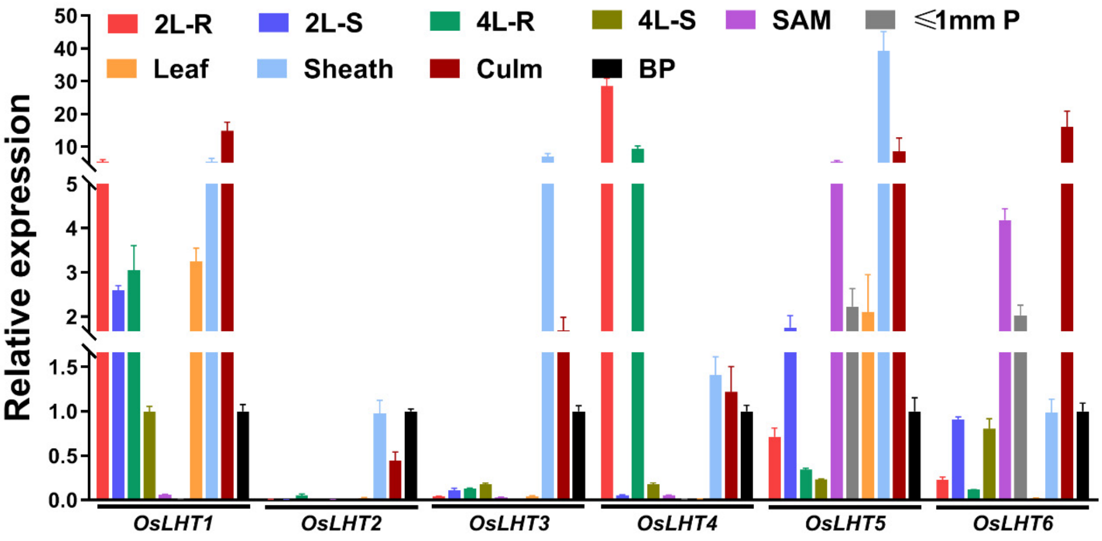

Figure S4. Expression patterns of 6 *OsLHT* genes under various tissues, as revealed by qRT-PCR.

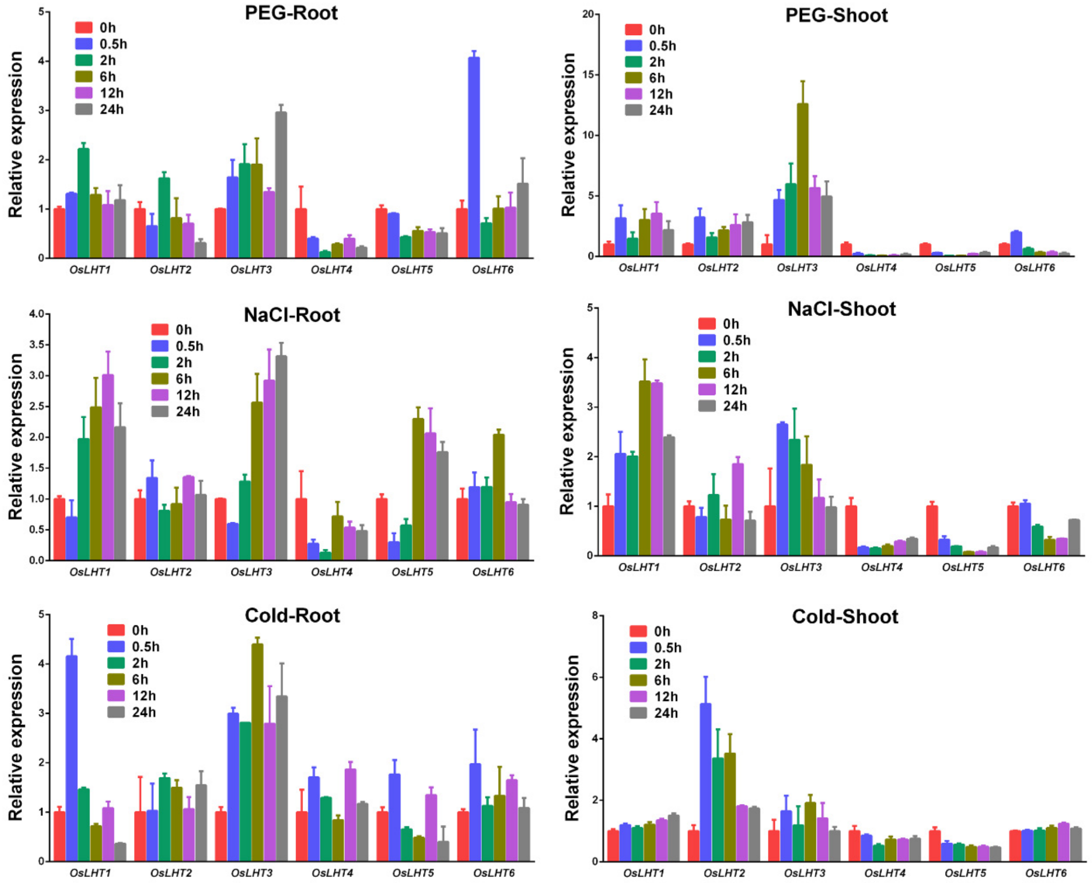

Figure S5. Expression patterns of 6 *OsLHT* genes under PEG, Cold and NaCl stress, as revealed by qRT-PCR.

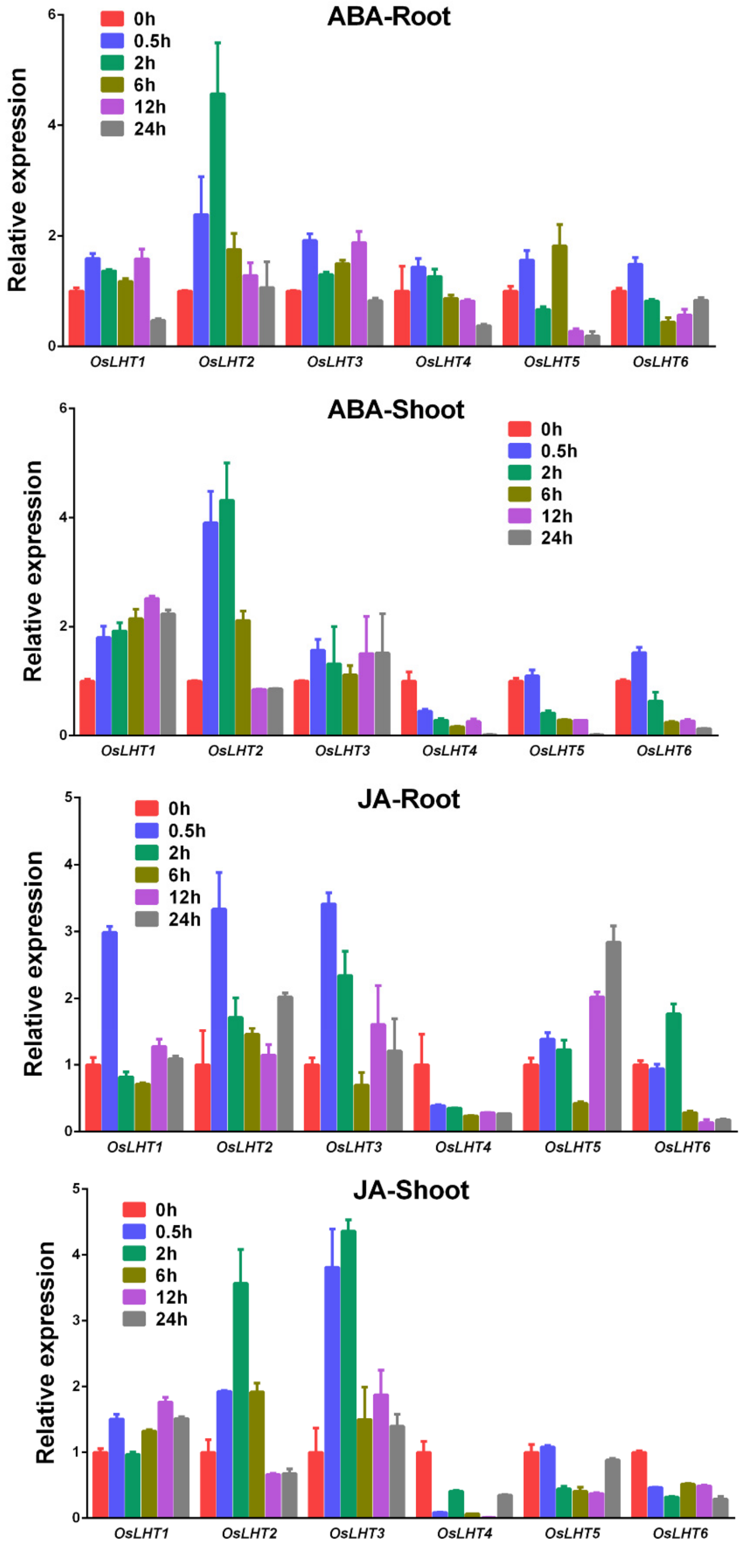

Figure S6. Expression patterns of 6 *OsLHT* genes under MeJA and ABA stress, as revealed by qRT-PCR.

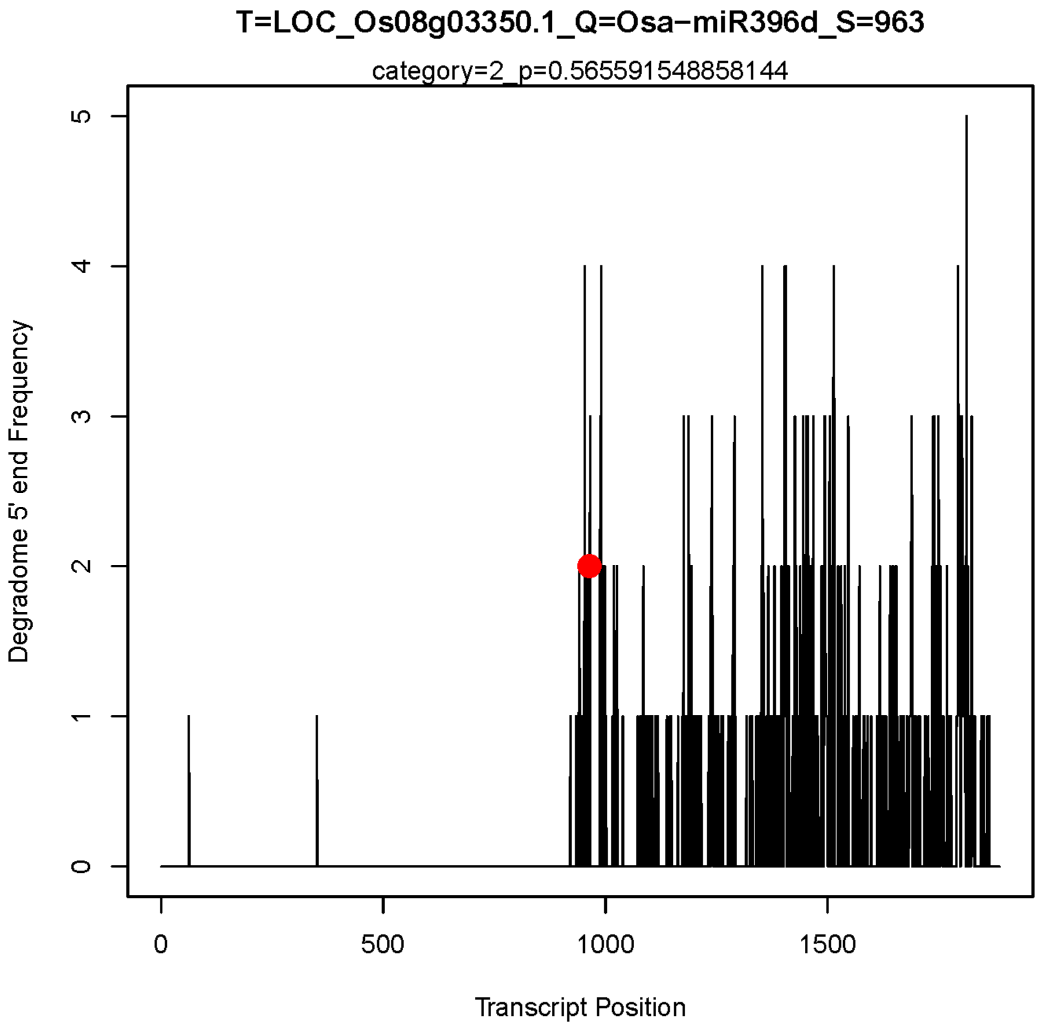

Figure S7. The degradome evidence of interaction between *OsLHT1* and miR396 from PmiREN.

Table S1. List of the primers in this study.

qRT-PCR primers

Name	Sequences (5'-3')
eEF-1α-F	GCACGCTCTTCTTGCTTTC
eEF-1α-R	AGGGAATCTTGTCAGGGTTG
*LHT1*q-F	GCCAACTACTTACTTCCTTCCCTG
*LHT1*q-R	CTGCCGGAGTCCTCCGATT
*LHT2*q-F	CGGAGGAGAAGGCCATCGA
*LHT2*q-R	AAGGGGAGGCTGAGGACGC
*LHT3*q-F	CGGAAGTGATGGAGGAGTGC
*LHT3*q-R	GCCACAGCGTGTAGAGCGT
*LHT4*q-F	CGGAAGTGATGGAGGAGTGC
*LHT4*q-R	GCCACAGCGTGTAGAGCGT
*LHT5*q-F	CCCAGCAAACGCACCAAAC
*LHT5*q-R	CTCACCCAGGTAGCCACTCA
*LHT6*q-F	GTCACCTTCGCCTACCCGT
*LHT6*q-R	GCTCCAGACACCACCCACA

Vector construction primers

Name	Sequences (5'-3')
*LHT1*GFP-F	gagctgcagaagcttactagtATGGGGACTCAGGTGGCAG
*LHT1*GFP-R	tccacttccacctccggtaccCGAGTAGAACTTGTATGTCTTGGC
*LHT2*GFP-F	gagctgcagaagcttactagtATGGCGCCGCCGTCAGCC
*LHT2*GFP-R	tccacttccacctccggtaccAGAGAAGAACTTGTAACTCTGGGC
*LHT3*GFP-F	gagctgcagaagcttactagtATGTCGGCGACGGAAGTGA
*LHT3*GFP-R	tccacttccacctccggtaccAGAGTAAAACTTGTATTTGCTAGCGTC
*LHT4*GFP-F	gagctgcagaagcttactagtATGGTCACTTCTTCAGTTCTTCCTAA
*LHT4*GFP-R	tccacttccacctccggtaccCGAGTAGAACTGGAACGTCGAGG
*LHT5*GFP-F	gagctgcagaagcttactagtATGTCGAGCGAGGTGACGTC
*LHT5*GFP-R	tccacttccacctccggtaccCTGGAAATCCGCAGGCTTG
*LHT6*GFP-F	gagctgcagaagcttactagtATGGCGATGGCGGTGGAG
*LHT6*GFP-R	Tccacttccacctccggtacc GCTTGGGGGCTTGAAGAACT
LHT1-Yeast-F	ATTCTCGAGATGGGGACTCAGGTGGCAG

LHT1-Yeast-R	CGCGGATCCCTACGAGTAGAACTTGTATG
LHT2-Yeast-F	ATTGTCGACATGGCGCCGCCGTCAG
LHT2-Yeast-R	CGCGGATCCTCAAGAGAAGAACTTG
LHT3-Yeast-F	TATCTCGAGATGTCGGCGACGGAAGTG
LHT3-Yeast-R	CGCGGATCCCTAAGAGTAAAACTTGT
LHT4-Yeast-F	AGCGTCGACATGGTCACTTCTTCAGT
LHT4-Yeast-R	ATCGGATCCTCACGAGTAGAACTGG
LHT5-Yeast-F	TATCTCGAGATGTCGAGCGAGGTGACGTCG
LHT5-Yeast-R	CGCGGATCCTCACTGGAAATCCGCAGGC
LHT6-Yeast-F	TATCTCGAGATGGCGATGGCGGTGGAG
LHT6-Yeast-R	AGTGGATCCCTAGCTTGGGGGCTTG

Table S2. The detailed functional annotations of cis-elements in promoter region of *OsLHT*.

Gene Name	Gene ID	Locus	Strands	Cis-Element	Sequences	Functional Annotations
OsLHT1	LOC_Os08g03350	1760	+	CCAAT-box	CAACGG	MYBHv1 binding site
OsLHT1	LOC_Os08g03350	93	+	AuxRR-core	GGTCCAT	cis-acting regulatory element involved in auxin responsiveness
OsLHT1	LOC_Os08g03350	1801	-	ABRE	ACGTG	cis-acting element involved in the abscisic acid responsiveness
OsLHT1	LOC_Os08g03350	1035	-	TC-rich repeats	GTTTTCTTAC	cis-acting element involved in defense and stress responsiveness
OsLHT1	LOC_Os08g03350	1399	+	TC-rich repeats	GTTTTCTTAC	cis-acting element involved in defense and stress responsiveness
OsLHT1	LOC_Os08g03350	402	-	G-box	CACGAC	cis-acting regulatory element involved in light responsiveness
OsLHT1	LOC_Os08g03350	1466	-	LTR	CCGAAA	cis-acting element involved in low-temperature responsiveness
OsLHT1	LOC_Os08g03350	1318	-	chs-CMA2b	GAACCTACACAC	part of a light responsive element
OsLHT1	LOC_Os08g03350	1274	+	A-box	CCGTCC	cis-acting regulatory element
OsLHT1	LOC_Os08g03350	1646	-	A-box	CCGTCC	cis-acting regulatory element
OsLHT1	LOC_Os08g03350	1403	+	TCT-motif	TCTTAC	part of a light responsive element
OsLHT1	LOC_Os08g03350	824	+	CAT-box	GCCACT	cis-acting regulatory element related to meristem expression
OsLHT1	LOC_Os08g03350	1372	+	TGACG-motif	TGACG	cis-acting regulatory element involved in the MeJA-responsiveness

OsLHT1	LOC_Os08g03350	1642	+	TGACG-motif	TGACG	cis-acting regulatory element involved in the MeJA-responsiveness
OsLHT1	LOC_Os08g03350	1801	+	G-Box	CACGTT	cis-acting regulatory element involved in light responsiveness
OsLHT1	LOC_Os08g03350	171	+	TATA-box	ATTATA	core promoter element around -30 of transcription start
OsLHT1	LOC_Os08g03350	172	-	TATA-box	TATAA	core promoter element around -30 of transcription start
OsLHT1	LOC_Os08g03350	173	+	TATA-box	TATA	core promoter element around -30 of transcription start
OsLHT1	LOC_Os08g03350	429	-	TATA-box	TATACA	core promoter element around -30 of transcription start
OsLHT1	LOC_Os08g03350	431	+	TATA-box	TATA	core promoter element around -30 of transcription start
OsLHT1	LOC_Os08g03350	515	+	TATA-box	TATA	core promoter element around -30 of transcription start
OsLHT1	LOC_Os08g03350	549	+	TATA-box	ATTATA	core promoter element around -30 of transcription start
OsLHT1	LOC_Os08g03350	550	-	TATA-box	TATAA	core promoter element around -30 of transcription start
OsLHT1	LOC_Os08g03350	551	+	TATA-box	TATA	core promoter element around -30 of transcription start
OsLHT1	LOC_Os08g03350	646	+	TATA-box	ATATAT	core promoter element around -30 of transcription start
OsLHT1	LOC_Os08g03350	647	+	TATA-box	TATA	core promoter element around -30 of transcription start
OsLHT1	LOC_Os08g03350	656	-	TATA-box	TATACA	core promoter element around -30 of transcription start
OsLHT1	LOC_Os08g03350	658	+	TATA-box	TATA	core promoter element around -30 of transcription start
OsLHT1	LOC_Os08g03350	746	+	TATA-box	ATATAT	core promoter element around -30 of transcription start
OsLHT1	LOC_Os08g03350	747	+	TATA-box	TATA	core promoter element around -30 of transcription start
OsLHT1	LOC_Os08g03350	775	-	TATA-box	TATACA	core promoter element around -30 of transcription start
OsLHT1	LOC_Os08g03350	777	+	TATA-box	TATA	core promoter element around -30 of transcription start

OsLHT1	LOC_Os08g03350	829	-	TATA-box	TACAAAA	core promoter element around -30 of transcription start
OsLHT1	LOC_Os08g03350	845	+	TATA-box	ATATAA	core promoter element around -30 of transcription start
OsLHT1	LOC_Os08g03350	846	+	TATA-box	TATA	core promoter element around -30 of transcription start
OsLHT1	LOC_Os08g03350	1086	-	TATA-box	TACATAAA	core promoter element around -30 of transcription start
OsLHT1	LOC_Os08g03350	1143	-	TATA-box	TATACA	core promoter element around -30 of transcription start
OsLHT1	LOC_Os08g03350	1145	-	TATA-box	TATA	core promoter element around -30 of transcription start
OsLHT1	LOC_Os08g03350	1226	-	TATA-box	TATA	core promoter element around -30 of transcription start
OsLHT1	LOC_Os08g03350	1278	+	TATA-box	ccTATAAAaa	core promoter element around -30 of transcription start
OsLHT1	LOC_Os08g03350	1349	-	TATA-box	ccTATAAAaa	core promoter element around -30 of transcription start
OsLHT1	LOC_Os08g03350	1350	-	TATA-box	TATAAAA	core promoter element around -30 of transcription start
OsLHT1	LOC_Os08g03350	1351	-	TATA-box	TATAAA	core promoter element around -30 of transcription start
OsLHT1	LOC_Os08g03350	1352	-	TATA-box	TATAA	core promoter element around -30 of transcription start
OsLHT1	LOC_Os08g03350	1353	-	TATA-box	TATA	core promoter element around -30 of transcription start
OsLHT1	LOC_Os08g03350	1379	-	TATA-box	TATA	core promoter element around -30 of transcription start
OsLHT1	LOC_Os08g03350	1487	-	TATA-box	TATAAA	core promoter element around -30 of transcription start
OsLHT1	LOC_Os08g03350	1488	-	TATA-box	TATAA	core promoter element around -30 of transcription start
OsLHT1	LOC_Os08g03350	1489	-	TATA-box	TATA	core promoter element around -30 of transcription start
OsLHT1	LOC_Os08g03350	1856	-	TATA-box	TATA	core promoter element around -30 of transcription start
OsLHT1	LOC_Os08g03350	196	-	ARE	AAACCA	cis-acting regulatory element essential for the anaerobic induction
OsLHT1	LOC_Os08g03350	1111	-	ATCT-motif	AATCTAATCC	part of a conserved DNA module involved in light responsiveness

OsLHT1	LOC_Os08g03350	1287	+	ATCT-motif	AATCTAATCC	part of a conserved DNA module involved in light responsiveness
OsLHT1	LOC_Os08g03350	1701	-	Box 4	ATTAAT	part of a conserved DNA module involved in light responsiveness
OsLHT1	LOC_Os08g03350	1372	-	CGTCA-motif	CGTCA	cis-acting regulatory element involved in the MeJA-responsiveness
OsLHT1	LOC_Os08g03350	1642	-	CGTCA-motif	CGTCA	cis-acting regulatory element involved in the MeJA-responsiveness
OsLHT1	LOC_Os08g03350	41	-	CAAT-box	CAAT	common cis-acting element in promoter and enhancer regions
OsLHT1	LOC_Os08g03350	137	-	CAAT-box	CAAAT	common cis-acting element in promoter and enhancer regions
OsLHT1	LOC_Os08g03350	187	+	CAAT-box	CAAAT	common cis-acting element in promoter and enhancer regions
OsLHT1	LOC_Os08g03350	274	+	CAAT-box	CAAT	common cis-acting element in promoter and enhancer regions
OsLHT1	LOC_Os08g03350	301	-	CAAT-box	CAAT	common cis-acting element in promoter and enhancer regions
OsLHT1	LOC_Os08g03350	320	-	CAAT-box	CAAAT	common cis-acting element in promoter and enhancer regions
OsLHT1	LOC_Os08g03350	338	-	CAAT-box	CAAT	common cis-acting element in promoter and enhancer regions
OsLHT1	LOC_Os08g03350	375	-	CAAT-box	CAAAT	common cis-acting element in promoter and enhancer regions
OsLHT1	LOC_Os08g03350	409	+	CAAT-box	CAAT	common cis-acting element in promoter and enhancer regions
OsLHT1	LOC_Os08g03350	438	-	CAAT-box	CAAAT	common cis-acting element in promoter and enhancer regions
OsLHT1	LOC_Os08g03350	459	-	CAAT-box	CAAT	common cis-acting element in promoter and enhancer regions
OsLHT1	LOC_Os08g03350	565	+	CAAT-box	CCAAT	common cis-acting element in promoter and enhancer regions
OsLHT1	LOC_Os08g03350	566	+	CAAT-box	CAAT	common cis-acting element in promoter and enhancer regions
OsLHT1	LOC_Os08g03350	602	+	CAAT-box	CAAAT	common cis-acting element in promoter and enhancer regions

OsLHT1	LOC_Os08g03350	618	+	CAAT-box	CAAT	common cis-acting element in promoter and enhancer regions
OsLHT1	LOC_Os08g03350	620	-	CAAT-box	CAAT	common cis-acting element in promoter and enhancer regions
OsLHT1	LOC_Os08g03350	635	-	CAAT-box	CAAT	common cis-acting element in promoter and enhancer regions
OsLHT1	LOC_Os08g03350	670	-	CAAT-box	TGCCAAC	common cis-acting element in promoter and enhancer regions
OsLHT1	LOC_Os08g03350	730	-	CAAT-box	CCAAT	common cis-acting element in promoter and enhancer regions
OsLHT1	LOC_Os08g03350	755	-	CAAT-box	CAAAT	common cis-acting element in promoter and enhancer regions
OsLHT1	LOC_Os08g03350	787	-	CAAT-box	CAAAT	common cis-acting element in promoter and enhancer regions
OsLHT1	LOC_Os08g03350	843	+	CAAT-box	CAAT	common cis-acting element in promoter and enhancer regions
OsLHT1	LOC_Os08g03350	866	-	CAAT-box	CAAT	common cis-acting element in promoter and enhancer regions
OsLHT1	LOC_Os08g03350	884	+	CAAT-box	CAAT	common cis-acting element in promoter and enhancer regions
OsLHT1	LOC_Os08g03350	916	-	CAAT-box	CAAAT	common cis-acting element in promoter and enhancer regions
OsLHT1	LOC_Os08g03350	923	-	CAAT-box	CCAAT	common cis-acting element in promoter and enhancer regions
OsLHT1	LOC_Os08g03350	951	-	CAAT-box	CAAAT	common cis-acting element in promoter and enhancer regions
OsLHT1	LOC_Os08g03350	993	+	CAAT-box	CAAT	common cis-acting element in promoter and enhancer regions
OsLHT1	LOC_Os08g03350	1099	+	CAAT-box	CAAAT	common cis-acting element in promoter and enhancer regions
OsLHT1	LOC_Os08g03350	1118	-	CAAT-box	CAAT	common cis-acting element in promoter and enhancer regions
OsLHT1	LOC_Os08g03350	1158	+	CAAT-box	CAAT	common cis-acting element in promoter and enhancer regions
OsLHT1	LOC_Os08g03350	1339	-	CAAT-box	CAAT	common cis-acting element in promoter and enhancer regions
OsLHT1	LOC_Os08g03350	1713	+	CAAT-box	CCAAT	common cis-acting element in promoter and enhancer regions
OsLHT1	LOC_Os08g03350	1714	+	CAAT-box	CAAT	common cis-acting element in promoter and enhancer regions

OsLHT1	LOC_Os08g03350	1784	-	CAAT-box	CAAT	common cis-acting element in promoter and enhancer regions
OsLHT1	LOC_Os08g03350	1949	-	CAAT-box	CAAT	common cis-acting element in promoter and enhancer regions
OsLHT1	LOC_Os08g03350	67	-	MBS	CAACTG	MYB binding site involved in drought-inducibility
OsLHT1	LOC_Os08g03350	702	+	MBS	CAACTG	MYB binding site involved in drought-inducibility
OsLHT1	LOC_Os08g03350	980	+	MBS	CAACTG	MYB binding site involved in drought-inducibility
OsLHT2	LOC_Os12g14100	246	+	TATA-box	TATA	core promoter element around -30 of transcription start
OsLHT2	LOC_Os12g14100	434	-	TATA-box	TATAAATA	core promoter element around -30 of transcription start
OsLHT2	LOC_Os12g14100	435	-	TATA-box	TATAAAT	core promoter element around -30 of transcription start
OsLHT2	LOC_Os12g14100	436	-	TATA-box	TATAAA	core promoter element around -30 of transcription start
OsLHT2	LOC_Os12g14100	437	-	TATA-box	TATAA	core promoter element around -30 of transcription start
OsLHT2	LOC_Os12g14100	438	+	TATA-box	TATA	core promoter element around -30 of transcription start
OsLHT2	LOC_Os12g14100	481	-	TATA-box	TATACA	core promoter element around -30 of transcription start
OsLHT2	LOC_Os12g14100	483	+	TATA-box	TATA	core promoter element around -30 of transcription start
OsLHT2	LOC_Os12g14100	694	+	TATA-box	TATA	core promoter element around -30 of transcription start
OsLHT2	LOC_Os12g14100	949	+	TATA-box	TATA	core promoter element around -30 of transcription start
OsLHT2	LOC_Os12g14100	1049	-	TATA-box	TATA	core promoter element around -30 of transcription start
OsLHT2	LOC_Os12g14100	1114	-	TATA-box	ATATAT	core promoter element around -30 of transcription start
OsLHT2	LOC_Os12g14100	1115	-	TATA-box	TATA	core promoter element around -30 of transcription start
OsLHT2	LOC_Os12g14100	1656	-	TATA-box	TATA	core promoter element around -30 of transcription start
OsLHT2	LOC_Os12g14100	1876	-	TATA-box	tcTATAAATAgg	core promoter element around -30 of transcription start

OsLHT2	LOC_Os12g14100	1878	-	TATA-box	TATAAATA	core promoter element around -30 of transcription start
OsLHT2	LOC_Os12g14100	1879	-	TATA-box	TATAAAT	core promoter element around -30 of transcription start
OsLHT2	LOC_Os12g14100	1880	-	TATA-box	TATAAA	core promoter element around -30 of transcription start
OsLHT2	LOC_Os12g14100	1881	-	TATA-box	TATAA	core promoter element around -30 of transcription start
OsLHT2	LOC_Os12g14100	1882	-	TATA-box	TATA	core promoter element around -30 of transcription start
OsLHT2	LOC_Os12g14100	1354	-	TCT-motif	TCTTAC	part of a light responsive element
OsLHT2	LOC_Os12g14100	230	+	ARE	AAACCA	cis-acting regulatory element essential for the anaerobic induction
OsLHT2	LOC_Os12g14100	647	-	ARE	AAACCA	cis-acting regulatory element essential for the anaerobic induction
OsLHT2	LOC_Os12g14100	935	+	ARE	AAACCA	cis-acting regulatory element essential for the anaerobic induction
OsLHT2	LOC_Os12g14100	117	-	CAAT-box	CCAAT	common cis-acting element in promoter and enhancer regions
OsLHT2	LOC_Os12g14100	158	-	CAAT-box	CAAT	common cis-acting element in promoter and enhancer regions
OsLHT2	LOC_Os12g14100	173	-	CAAT-box	CAAT	common cis-acting element in promoter and enhancer regions
OsLHT2	LOC_Os12g14100	183	-	CAAT-box	CAAT	common cis-acting element in promoter and enhancer regions
OsLHT2	LOC_Os12g14100	394	-	CAAT-box	CAACCAACTCC	common cis-acting element in promoter and enhancer regions
OsLHT2	LOC_Os12g14100	478	-	CAAT-box	CAAAT	common cis-acting element in promoter and enhancer regions
OsLHT2	LOC_Os12g14100	581	-	CAAT-box	CAAAT	common cis-acting element in promoter and enhancer regions
OsLHT2	LOC_Os12g14100	617	+	CAAT-box	CAAT	common cis-acting element in promoter and enhancer regions
OsLHT2	LOC_Os12g14100	619	-	CAAT-box	CAAT	common cis-acting element in promoter and enhancer regions
OsLHT2	LOC_Os12g14100	701	+	CAAT-box	CAAT	common cis-acting element in promoter and enhancer regions
OsLHT2	LOC_Os12g14100	727	+	CAAT-box	CAAT	common cis-acting element in promoter and enhancer regions

OsLHT2	LOC_Os12g14100	739	-	CAAT-box	CAAAT	common cis-acting element in promoter and enhancer regions
OsLHT2	LOC_Os12g14100	796	-	CAAT-box	CAAT	common cis-acting element in promoter and enhancer regions
OsLHT2	LOC_Os12g14100	886	+	CAAT-box	CCCAATTT	common cis-acting element in promoter and enhancer regions
OsLHT2	LOC_Os12g14100	887	+	CAAT-box	CCAAT	common cis-acting element in promoter and enhancer regions
OsLHT2	LOC_Os12g14100	888	+	CAAT-box	CAAT	common cis-acting element in promoter and enhancer regions
OsLHT2	LOC_Os12g14100	930	-	CAAT-box	CAAAT	common cis-acting element in promoter and enhancer regions
OsLHT2	LOC_Os12g14100	938	+	CAAT-box	CCAAT	common cis-acting element in promoter and enhancer regions
OsLHT2	LOC_Os12g14100	939	+	CAAT-box	CAAT	common cis-acting element in promoter and enhancer regions
OsLHT2	LOC_Os12g14100	1025	-	CAAT-box	CAAT	common cis-acting element in promoter and enhancer regions
OsLHT2	LOC_Os12g14100	1038	-	CAAT-box	CAAT	common cis-acting element in promoter and enhancer regions
OsLHT2	LOC_Os12g14100	1137	+	CAAT-box	CAAT	common cis-acting element in promoter and enhancer regions
OsLHT2	LOC_Os12g14100	1239	-	CAAT-box	CAAAT	common cis-acting element in promoter and enhancer regions
OsLHT2	LOC_Os12g14100	1474	+	CAAT-box	CAAAT	common cis-acting element in promoter and enhancer regions
OsLHT2	LOC_Os12g14100	1524	+	CAAT-box	CAAAT	common cis-acting element in promoter and enhancer regions
OsLHT2	LOC_Os12g14100	1549	-	CAAT-box	CAAAT	common cis-acting element in promoter and enhancer regions
OsLHT2	LOC_Os12g14100	1586	+	CAAT-box	CCAAT	common cis-acting element in promoter and enhancer regions
OsLHT2	LOC_Os12g14100	1587	+	CAAT-box	CAAT	common cis-acting element in promoter and enhancer regions
OsLHT2	LOC_Os12g14100	1964	+	TCCC-motif	TCTCCCT	part of a light responsive element
OsLHT2	LOC_Os12g14100	591	+	GT1-motif	GGTTAA	light responsive element
OsLHT2	LOC_Os12g14100	804	-	GT1-motif	GGTTAA	light responsive element

OsLHT2	LOC_Os12g14100	1581	-	GT1-motif	GGTTAA	light responsive element
OsLHT2	LOC_Os12g14100	639	+	I-box	gGATAAGGTG	part of a light responsive element
OsLHT2	LOC_Os12g14100	571	+	GATA-motif	GATAGGA	part of a light responsive element
OsLHT2	LOC_Os12g14100	1275	+	GATA-motif	GATAGGA	part of a light responsive element
OsLHT2	LOC_Os12g14100	1619	+	GATA-motif	AAGATAAGATT	part of a light responsive element
OsLHT2	LOC_Os12g14100	1145	+	AuxRR-core	GGTCCAT	cis-acting regulatory element involved in auxin responsiveness
OsLHT2	LOC_Os12g14100	1345	+	P-box	CCTTTTG	gibberellin-responsive element
OsLHT2	LOC_Os12g14100	809	+	circadian	CAAAGATATC	cis-acting regulatory element involved in circadian control
OsLHT2	LOC_Os12g14100	101	+	ABRE	CACGTG	cis-acting element involved in the abscisic acid responsiveness
OsLHT2	LOC_Os12g14100	102	+	ABRE	ACGTG	cis-acting element involved in the abscisic acid responsiveness
OsLHT3	LOC_Os05g14820	1036	+	O2-site	GATGACATGG	cis-acting regulatory element involved in zein metabolism regulation
OsLHT3	LOC_Os05g14820	1460	-	LTR	CCGAAA	cis-acting element involved in low-temperature responsiveness
OsLHT3	LOC_Os05g14820	1467	+	LTR	CCGAAA	cis-acting element involved in low-temperature responsiveness
OsLHT3	LOC_Os05g14820	1605	+	LTR	CCGAAA	cis-acting element involved in low-temperature responsiveness
OsLHT3	LOC_Os05g14820	280	+	CAT-box	GCCACT	cis-acting regulatory element related to meristem expression
OsLHT3	LOC_Os05g14820	1765	+	GT1-motif	GGTTAA	light responsive element
OsLHT3	LOC_Os05g14820	212	-	G-box	CAGACGTGGCA	cis-acting regulatory element involved in light responsiveness
OsLHT3	LOC_Os05g14820	215	+	G-box	CACGTC	cis-acting regulatory element involved in light responsiveness
OsLHT3	LOC_Os05g14820	1138	+	G-box	CACGTC	cis-acting regulatory element involved in light responsiveness

OsLHT3	LOC_Os05g14820	1168	-	G-box	TACGTG	cis-acting regulatory element involved in light responsiveness
OsLHT3	LOC_Os05g14820	1179	+	G-box	CACGTC	cis-acting regulatory element involved in light responsiveness
OsLHT3	LOC_Os05g14820	293	+	TGACG-motif	TGACG	cis-acting regulatory element involved in the MeJA-responsiveness
OsLHT3	LOC_Os05g14820	344	+	TGACG-motif	TGACG	cis-acting regulatory element involved in the MeJA-responsiveness
OsLHT3	LOC_Os05g14820	383	-	TGACG-motif	TGACG	cis-acting regulatory element involved in the MeJA-responsiveness
OsLHT3	LOC_Os05g14820	435	-	TGACG-motif	TGACG	cis-acting regulatory element involved in the MeJA-responsiveness
OsLHT3	LOC_Os05g14820	520	-	TGACG-motif	TGACG	cis-acting regulatory element involved in the MeJA-responsiveness
OsLHT3	LOC_Os05g14820	625	-	TGACG-motif	TGACG	cis-acting regulatory element involved in the MeJA-responsiveness
OsLHT3	LOC_Os05g14820	1140	-	TGACG-motif	TGACG	cis-acting regulatory element involved in the MeJA-responsiveness
OsLHT3	LOC_Os05g14820	1181	-	TGACG-motif	TGACG	cis-acting regulatory element involved in the MeJA-responsiveness
OsLHT3	LOC_Os05g14820	22	-	I-box	gGATAAGGTG	part of a light responsive element
OsLHT3	LOC_Os05g14820	24	+	I-box	ccttatcct	part of a light responsive element

OsLHT3	LOC_Os05g14820	368	+	CAAT-box	CAACCAACTCC	common cis-acting element in promoter and enhancer regions
OsLHT3	LOC_Os05g14820	460	+	CAAT-box	CAAAT	common cis-acting element in promoter and enhancer regions
OsLHT3	LOC_Os05g14820	473	+	CAAT-box	TGCCAAC	common cis-acting element in promoter and enhancer regions
OsLHT3	LOC_Os05g14820	1030	-	CAAT-box	CAAT	common cis-acting element in promoter and enhancer regions
OsLHT3	LOC_Os05g14820	1122	-	CAAT-box	CAAT	common cis-acting element in promoter and enhancer regions
OsLHT3	LOC_Os05g14820	1159	+	CAAT-box	CAAAT	common cis-acting element in promoter and enhancer regions
OsLHT3	LOC_Os05g14820	1239	-	CAAT-box	CAAT	common cis-acting element in promoter and enhancer regions
OsLHT3	LOC_Os05g14820	1371	-	CAAT-box	CCAAT	common cis-acting element in promoter and enhancer regions
OsLHT3	LOC_Os05g14820	1788	+	CAAT-box	CAAT	common cis-acting element in promoter and enhancer regions
OsLHT3	LOC_Os05g14820	810	-	Sp1	GGGCGG	light responsive element
OsLHT3	LOC_Os05g14820	1632	-	ACE	GCGACGTACC	cis-acting element involved in light responsiveness
OsLHT3	LOC_Os05g14820	293	-	CGTCA-motif	CGTCA	cis-acting regulatory element involved in the MeJA-responsiveness
OsLHT3	LOC_Os05g14820	344	-	CGTCA-motif	CGTCA	cis-acting regulatory element involved in the MeJA-responsiveness
OsLHT3	LOC_Os05g14820	383	+	CGTCA-motif	CGTCA	cis-acting regulatory element involved in the MeJA-responsiveness
OsLHT3	LOC_Os05g14820	435	+	CGTCA-motif	CGTCA	cis-acting regulatory element involved in the MeJA-responsiveness

OsLHT3	LOC_Os05g14820	520	+	CGTCA-motif	CGTCA	cis-acting regulatory element involved in the MeJA-responsiveness
OsLHT3	LOC_Os05g14820	625	+	CGTCA-motif	CGTCA	cis-acting regulatory element involved in the MeJA-responsiveness
OsLHT3	LOC_Os05g14820	1140	+	CGTCA-motif	CGTCA	cis-acting regulatory element involved in the MeJA-responsiveness
OsLHT3	LOC_Os05g14820	1181	+	CGTCA-motif	CGTCA	cis-acting regulatory element involved in the MeJA-responsiveness
OsLHT3	LOC_Os05g14820	1259	-	ARE	AAACCA	cis-acting regulatory element essential for the anaerobic induction
OsLHT3	LOC_Os05g14820	1471	+	ARE	AAACCA	cis-acting regulatory element essential for the anaerobic induction
OsLHT3	LOC_Os05g14820	215	-	ABRE	ACGTG	cis-acting element involved in the abscisic acid responsiveness
OsLHT3	LOC_Os05g14820	600	+	ABRE	CGCACGTGTC	cis-acting element involved in the abscisic acid responsiveness
OsLHT3	LOC_Os05g14820	1138	-	ABRE	ACGTG	cis-acting element involved in the abscisic acid responsiveness
OsLHT3	LOC_Os05g14820	1168	-	ABRE	ACGTG	cis-acting element involved in the abscisic acid responsiveness
OsLHT3	LOC_Os05g14820	1179	-	ABRE	ACGTG	cis-acting element involved in the abscisic acid responsiveness
OsLHT3	LOC_Os05g14820	24	-	GATA-motif	AAGGATAAGG	part of a light responsive element
OsLHT3	LOC_Os05g14820	645	-	GATA-motif	AAGGATAAGG	part of a light responsive element
OsLHT3	LOC_Os05g14820	390	-	TATA-box	TATAA	core promoter element around -30 of transcription start
OsLHT3	LOC_Os05g14820	391	+	TATA-box	TATA	core promoter element around -30 of transcription start
OsLHT3	LOC_Os05g14820	1526	+	TATA-box	TACAAAA	core promoter element around -30 of transcription start

OsLHT3	LOC_Os05g14820	1697	+	TATA-box	ATATAA	core promoter element around -30 of transcription start
OsLHT3	LOC_Os05g14820	1698	-	TATA-box	TATA	core promoter element around -30 of transcription start
OsLHT3	LOC_Os05g14820	1889	-	TATA-box	TATATA	core promoter element around -30 of transcription start
OsLHT3	LOC_Os05g14820	1890	-	TATA-box	ATATAT	core promoter element around -30 of transcription start
OsLHT3	LOC_Os05g14820	1891	-	TATA-box	TATATA	core promoter element around -30 of transcription start
OsLHT3	LOC_Os05g14820	1892	-	TATA-box	ATATAT	core promoter element around -30 of transcription start
OsLHT3	LOC_Os05g14820	1893	-	TATA-box	TATA	core promoter element around -30 of transcription start
OsLHT4	LOC_Os04g38860	697	+	A-box	CCGTCC	cis-acting regulatory element
OsLHT4	LOC_Os04g38860	290	+	TCCC-motif	TCTCCCT	part of a light responsive element
OsLHT4	LOC_Os04g38860	644	+	TCCC-motif	TCTCCCT	part of a light responsive element
OsLHT4	LOC_Os04g38860	923	-	TCCC-motif	TCTCCCT	part of a light responsive element
OsLHT4	LOC_Os04g38860	173	-	G-box	CACGTC	cis-acting regulatory element involved in light responsiveness
OsLHT4	LOC_Os04g38860	1033	+	G-box	CCACGTAA	cis-acting regulatory element involved in light responsiveness
OsLHT4	LOC_Os04g38860	1034	-	G-box	TACGTG	cis-acting regulatory element involved in light responsiveness
OsLHT4	LOC_Os04g38860	1043	+	G-box	GCCACGTGGA	cis-acting regulatory element involved in light responsiveness
OsLHT4	LOC_Os04g38860	1045	-	G-box	CACGTG	cis-acting regulatory element involved in light responsiveness
OsLHT4	LOC_Os04g38860	1075	+	G-box	CACGTC	cis-acting regulatory element involved in light responsiveness
OsLHT4	LOC_Os04g38860	1407	-	G-box	CACGAC	cis-acting regulatory element involved in light responsiveness
OsLHT4	LOC_Os04g38860	172	+	TGACG-motif	TGACG	cis-acting regulatory element involved in the MeJA-responsiveness

OsLHT4	LOC_Os04g38860	492	+	TGACG-motif	TGACG	cis-acting regulatory element involved in the MeJA-responsiveness
OsLHT4	LOC_Os04g38860	1077	-	TGACG-motif	TGACG	cis-acting regulatory element involved in the MeJA-responsiveness
OsLHT4	LOC_Os04g38860	1356	-	TGACG-motif	TGACG	cis-acting regulatory element involved in the MeJA-responsiveness
OsLHT4	LOC_Os04g38860	1364	-	TGACG-motif	TGACG	cis-acting regulatory element involved in the MeJA-responsiveness
OsLHT4	LOC_Os04g38860	118	+	O2-site	GATGATGTGG	cis-acting regulatory element involved in zein metabolism regulation
OsLHT4	LOC_Os04g38860	170	+	O2-site	GTTGACGTGA	cis-acting regulatory element involved in zein metabolism regulation
OsLHT4	LOC_Os04g38860	264	-	O2-site	GATGACATGG	cis-acting regulatory element involved in zein metabolism regulation
OsLHT4	LOC_Os04g38860	939	+	O2-site	GATGACATGG	cis-acting regulatory element involved in zein metabolism regulation
OsLHT4	LOC_Os04g38860	48	+	TATA-box	ATATAA	core promoter element around -30 of transcription start
OsLHT4	LOC_Os04g38860	49	+	TATA-box	TATA	core promoter element around -30 of transcription start
OsLHT4	LOC_Os04g38860	196	+	TATA-box	ATATAT	core promoter element around -30 of transcription start
OsLHT4	LOC_Os04g38860	197	+	TATA-box	TATA	core promoter element around -30 of transcription start

OsLHT4	LOC_Os04g38860	232	+	TATA-box	ATATAT	core promoter element around -30 of transcription start
OsLHT4	LOC_Os04g38860	233	+	TATA-box	TATA	core promoter element around -30 of transcription start
OsLHT4	LOC_Os04g38860	1011	-	TATA-box	ATATAT	core promoter element around -30 of transcription start
OsLHT4	LOC_Os04g38860	1012	-	TATA-box	TATA	core promoter element around -30 of transcription start
OsLHT4	LOC_Os04g38860	1946	-	TATA-box	TATA	core promoter element around -30 of transcription start
OsLHT4	LOC_Os04g38860	68	-	CAAT-box	CAAT	common cis-acting element in promoter and enhancer regions
OsLHT4	LOC_Os04g38860	81	+	CAAT-box	CAAT	common cis-acting element in promoter and enhancer regions
OsLHT4	LOC_Os04g38860	83	-	CAAT-box	CAAAT	common cis-acting element in promoter and enhancer regions
OsLHT4	LOC_Os04g38860	139	+	CAAT-box	CAAT	common cis-acting element in promoter and enhancer regions
OsLHT4	LOC_Os04g38860	236	-	CAAT-box	CAAT	common cis-acting element in promoter and enhancer regions
OsLHT4	LOC_Os04g38860	321	+	CAAT-box	CAAT	common cis-acting element in promoter and enhancer regions
OsLHT4	LOC_Os04g38860	555	-	CAAT-box	CAAT	common cis-acting element in promoter and enhancer regions
OsLHT4	LOC_Os04g38860	724	+	CAAT-box	CAAT	common cis-acting element in promoter and enhancer regions
OsLHT4	LOC_Os04g38860	773	+	CAAT-box	CAAT	common cis-acting element in promoter and enhancer regions
OsLHT4	LOC_Os04g38860	895	-	CAAT-box	CAAT	common cis-acting element in promoter and enhancer regions
OsLHT4	LOC_Os04g38860	962	+	CAAT-box	CAAT	common cis-acting element in promoter and enhancer regions
OsLHT4	LOC_Os04g38860	974	+	CAAT-box	CAAT	common cis-acting element in promoter and enhancer regions
OsLHT4	LOC_Os04g38860	1015	-	CAAT-box	CAAAT	common cis-acting element in promoter and enhancer regions
OsLHT4	LOC_Os04g38860	1125	+	CAAT-box	CAAAT	common cis-acting element in promoter and enhancer regions
OsLHT4	LOC_Os04g38860	1128	-	CAAT-box	CAAT	common cis-acting element in promoter and enhancer regions

OsLHT4	LOC_Os04g38860	1141	+	CAAT-box	CAAT	common cis-acting element in promoter and enhancer regions
OsLHT4	LOC_Os04g38860	1230	-	CAAT-box	CAAT	common cis-acting element in promoter and enhancer regions
OsLHT4	LOC_Os04g38860	1253	+	CAAT-box	CAAAT	common cis-acting element in promoter and enhancer regions
OsLHT4	LOC_Os04g38860	1286	+	CAAT-box	CCAAT	common cis-acting element in promoter and enhancer regions
OsLHT4	LOC_Os04g38860	1287	+	CAAT-box	CAAT	common cis-acting element in promoter and enhancer regions
OsLHT4	LOC_Os04g38860	1329	-	CAAT-box	CCAAT	common cis-acting element in promoter and enhancer regions
OsLHT4	LOC_Os04g38860	1359	+	CAAT-box	CAAT	common cis-acting element in promoter and enhancer regions
OsLHT4	LOC_Os04g38860	1542	-	CAAT-box	CAAT	common cis-acting element in promoter and enhancer regions
OsLHT4	LOC_Os04g38860	1555	-	CAAT-box	CAAT	common cis-acting element in promoter and enhancer regions
OsLHT4	LOC_Os04g38860	1595	+	CAAT-box	CAAAT	common cis-acting element in promoter and enhancer regions
OsLHT4	LOC_Os04g38860	1673	+	CAAT-box	CAAAT	common cis-acting element in promoter and enhancer regions
OsLHT4	LOC_Os04g38860	1679	-	CAAT-box	CAAT	common cis-acting element in promoter and enhancer regions
OsLHT4	LOC_Os04g38860	1701	-	CAAT-box	CAAT	common cis-acting element in promoter and enhancer regions
OsLHT4	LOC_Os04g38860	1818	+	CAAT-box	CCAAT	common cis-acting element in promoter and enhancer regions
OsLHT4	LOC_Os04g38860	1819	+	CAAT-box	CAAT	common cis-acting element in promoter and enhancer regions
OsLHT4	LOC_Os04g38860	1930	-	CAAT-box	CCAAT	common cis-acting element in promoter and enhancer regions
OsLHT4	LOC_Os04g38860	1523	-	LTR	CCGAAA	cis-acting element involved in low-temperature responsiveness
OsLHT4	LOC_Os04g38860	1805	-	LTR	CCGAAA	cis-acting element involved in low-temperature responsiveness
OsLHT4	LOC_Os04g38860	1159	-	GATA-motif	GATAGGA	part of a light responsive element
OsLHT4	LOC_Os04g38860	6	-	CAT-box	GCCACT	cis-acting regulatory element related to meristem expression

OsLHT4	LOC_Os04g38860	361	-	CAT-box	GCCACT	cis-acting regulatory element related to meristem expression
OsLHT4	LOC_Os04g38860	389	-	CAT-box	GCCACT	cis-acting regulatory element related to meristem expression
OsLHT4	LOC_Os04g38860	473	-	CAT-box	GCCACT	cis-acting regulatory element related to meristem expression
OsLHT4	LOC_Os04g38860	72	+	ARE	AAACCA	cis-acting regulatory element essential for the anaerobic induction
OsLHT4	LOC_Os04g38860	1135	-	ARE	AAACCA	cis-acting regulatory element essential for the anaerobic induction
OsLHT4	LOC_Os04g38860	1499	+	ARE	AAACCA	cis-acting regulatory element essential for the anaerobic induction
OsLHT4	LOC_Os04g38860	1669	+	ARE	AAACCA	cis-acting regulatory element essential for the anaerobic induction
OsLHT4	LOC_Os04g38860	1932	-	ARE	AAACCA	cis-acting regulatory element essential for the anaerobic induction
OsLHT4	LOC_Os04g38860	174	+	ABRE	ACGTG	cis-acting element involved in the abscisic acid responsiveness
OsLHT4	LOC_Os04g38860	1034	-	ABRE	ACGTG	cis-acting element involved in the abscisic acid responsiveness
OsLHT4	LOC_Os04g38860	1045	-	ABRE	CACGTG	cis-acting element involved in the abscisic acid responsiveness
OsLHT4	LOC_Os04g38860	1046	+	ABRE	ACGTG	cis-acting element involved in the abscisic acid responsiveness
OsLHT4	LOC_Os04g38860	1075	-	ABRE	ACGTG	cis-acting element involved in the abscisic acid responsiveness
OsLHT4	LOC_Os04g38860	1378	+	ABRE	CGCACGTGTC	cis-acting element involved in the abscisic acid responsiveness
OsLHT4	LOC_Os04g38860	1484	+	ABRE	ACGTG	cis-acting element involved in the abscisic acid responsiveness
OsLHT4	LOC_Os04g38860	108	+	Sp1	GGGCGG	light responsive element
OsLHT4	LOC_Os04g38860	335	+	Sp1	GGGCGG	light responsive element
OsLHT4	LOC_Os04g38860	348	+	Sp1	GGGCGG	light responsive element
OsLHT4	LOC_Os04g38860	623	-	Sp1	GGGCGG	light responsive element
OsLHT4	LOC_Os04g38860	671	-	Sp1	GGGCGG	light responsive element

OsLHT4	LOC_Os04g38860	879	-	Sp1	GGGCGG	light responsive element
OsLHT4	LOC_Os04g38860	172	-	CGTCA-motif	CGTCA	cis-acting regulatory element involved in the MeJA-responsiveness
OsLHT4	LOC_Os04g38860	492	-	CGTCA-motif	CGTCA	cis-acting regulatory element involved in the MeJA-responsiveness
OsLHT4	LOC_Os04g38860	1077	+	CGTCA-motif	CGTCA	cis-acting regulatory element involved in the MeJA-responsiveness
OsLHT4	LOC_Os04g38860	1356	+	CGTCA-motif	CGTCA	cis-acting regulatory element involved in the MeJA-responsiveness
OsLHT4	LOC_Os04g38860	1364	+	CGTCA-motif	CGTCA	cis-acting regulatory element involved in the MeJA-responsiveness
OsLHT4	LOC_Os04g38860	52	+	TGA-element	AACGAC	auxin-responsive element
OsLHT4	LOC_Os04g38860	15	+	ATCT-motif	AATCTAATCC	part of a conserved DNA module involved in light responsiveness
OsLHT4	LOC_Os04g38860	1188	-	ATCT-motif	AATCTAATCC	part of a conserved DNA module involved in light responsiveness
OsLHT4	LOC_Os04g38860	1630	+	ATCT-motif	AATCTAATCC	part of a conserved DNA module involved in light responsiveness
OsLHT4	LOC_Os04g38860	532	+	GC-motif	CCCCCG	enhancer-like element involved in anoxic specific inducibility
OsLHT4	LOC_Os04g38860	1458	+	MRE	AACCTAA	MYB binding site involved in light responsiveness
OsLHT4	LOC_Os04g38860	245	+	MBS	CAACTG	MYB binding site involved in drought-inducibility
OsLHT4	LOC_Os04g38860	1045	-	G-Box	CACGTG	cis-acting regulatory element involved in light responsiveness
OsLHT4	LOC_Os04g38860	1483	-	G-Box	CACGTT	cis-acting regulatory element involved in light responsiveness

OsLHT4	LOC_Os04g38860	1456	-	GT1-motif	GGTTAA	light responsive element
OsLHT4	LOC_Os04g38860	1747	+	P-box	CCTTTTG	gibberellin-responsive element
OsLHT4	LOC_Os04g38860	1798	+	P-box	CCTTTTG	gibberellin-responsive element
OsLHT4	LOC_Os04g38860	450	-	CCAAT-box	CAACGG	MYBHv1 binding site
OsLHT4	LOC_Os04g38860	1303	-	CCAAT-box	CAACGG	MYBHv1 binding site
OsLHT5	LOC_Os04g47420	326	-	CAAT-box	CAAAT	common cis-acting element in promoter and enhancer regions
OsLHT5	LOC_Os04g47420	364	+	CAAT-box	CAAAT	common cis-acting element in promoter and enhancer regions
OsLHT5	LOC_Os04g47420	376	+	CAAT-box	CAAT	common cis-acting element in promoter and enhancer regions
OsLHT5	LOC_Os04g47420	392	+	CAAT-box	CAAT	common cis-acting element in promoter and enhancer regions
OsLHT5	LOC_Os04g47420	397	+	CAAT-box	CAAT	common cis-acting element in promoter and enhancer regions
OsLHT5	LOC_Os04g47420	461	+	CAAT-box	CAAT	common cis-acting element in promoter and enhancer regions
OsLHT5	LOC_Os04g47420	487	+	CAAT-box	CAAT	common cis-acting element in promoter and enhancer regions
OsLHT5	LOC_Os04g47420	543	+	CAAT-box	CAAT	common cis-acting element in promoter and enhancer regions
OsLHT5	LOC_Os04g47420	560	+	CAAT-box	CAAT	common cis-acting element in promoter and enhancer regions
OsLHT5	LOC_Os04g47420	694	+	CAAT-box	CAAT	common cis-acting element in promoter and enhancer regions
OsLHT5	LOC_Os04g47420	1095	-	CAAT-box	CCAAT	common cis-acting element in promoter and enhancer regions
OsLHT5	LOC_Os04g47420	1187	-	CAAT-box	CAAT	common cis-acting element in promoter and enhancer regions
OsLHT5	LOC_Os04g47420	1285	-	CAAT-box	CAAAT	common cis-acting element in promoter and enhancer regions
OsLHT5	LOC_Os04g47420	1497	+	CAAT-box	CAAAT	common cis-acting element in promoter and enhancer regions
OsLHT5	LOC_Os04g47420	1559	+	CAAT-box	CAAAT	common cis-acting element in promoter and enhancer regions

OsLHT5	LOC_Os04g47420	1764	+	CAAT-box	CAAT	common cis-acting element in promoter and enhancer regions
OsLHT5	LOC_Os04g47420	702	-	ATCT-motif	AATCTAATCC	part of a conserved DNA module involved in light responsiveness
OsLHT5	LOC_Os04g47420	1108	-	TGA-element	AACGAC	auxin-responsive element
OsLHT5	LOC_Os04g47420	665	-	GC-motif	CCCCCG	enhancer-like element involved in anoxic specific inducibility
OsLHT5	LOC_Os04g47420	952	-	GC-motif	CGGCGCCCT	enhancer-like element involved in anoxic specific inducibility
OsLHT5	LOC_Os04g47420	540	+	CGTCA-motif	CGTCA	cis-acting regulatory element involved in the MeJA-responsiveness
OsLHT5	LOC_Os04g47420	784	-	CGTCA-motif	CGTCA	cis-acting regulatory element involved in the MeJA-responsiveness
OsLHT5	LOC_Os04g47420	969	+	CGTCA-motif	CGTCA	cis-acting regulatory element involved in the MeJA-responsiveness
OsLHT5	LOC_Os04g47420	1087	+	CGTCA-motif	CGTCA	cis-acting regulatory element involved in the MeJA-responsiveness
OsLHT5	LOC_Os04g47420	1602	-	ABRE	ACGTG	cis-acting element involved in the abscisic acid responsiveness
OsLHT5	LOC_Os04g47420	1684	+	ABRE	ACGTG	cis-acting element involved in the abscisic acid responsiveness
OsLHT5	LOC_Os04g47420	1742	+	ABRE	TACGGTC	cis-acting element involved in the abscisic acid responsiveness
OsLHT5	LOC_Os04g47420	584	-	AuxRR-core	GGTCCAT	cis-acting regulatory element involved in auxin responsiveness
OsLHT5	LOC_Os04g47420	1702	-	TCCC-motif	TCTCCCT	part of a light responsive element
OsLHT5	LOC_Os04g47420	1022	-	GATA-motif	GATAGGA	part of a light responsive element
OsLHT5	LOC_Os04g47420	1162	+	GATA-motif	GATAGGA	part of a light responsive element

OsLHT5	LOC_Os04g47420	182	-	O2-site	GATGATGTGG	cis-acting regulatory element involved in zein metabolism regulation
OsLHT5	LOC_Os04g47420	578	+	O2-site	GATGACATGG	cis-acting regulatory element involved in zein metabolism regulation
OsLHT5	LOC_Os04g47420	1479	-	MRE	AACCTAA	MYB binding site involved in light responsiveness
OsLHT5	LOC_Os04g47420	644	+	Box 4	ATTAAT	part of a conserved DNA module involved in light responsiveness
OsLHT5	LOC_Os04g47420	1585	-	Box 4	ATTAAT	part of a conserved DNA module involved in light responsiveness
OsLHT5	LOC_Os04g47420	1000	-	G-box	CACGAC	cis-acting regulatory element involved in light responsiveness
OsLHT5	LOC_Os04g47420	1602	-	G-box	TACGTG	cis-acting regulatory element involved in light responsiveness
OsLHT5	LOC_Os04g47420	1683	+	G-box	TACGTG	cis-acting regulatory element involved in light responsiveness
OsLHT5	LOC_Os04g47420	1746	-	G-box	CACGAC	cis-acting regulatory element involved in light responsiveness
OsLHT5	LOC_Os04g47420	16	+	CCAAT-box	CAACGG	MYBHv1 binding site
OsLHT5	LOC_Os04g47420	34	-	CCAAT-box	CAACGG	MYBHv1 binding site
OsLHT5	LOC_Os04g47420	615	+	CCAAT-box	CAACGG	MYBHv1 binding site
OsLHT5	LOC_Os04g47420	205	+	Sp1	GGGCGG	light responsive element
OsLHT5	LOC_Os04g47420	1074	-	Sp1	GGGCGG	light responsive element
OsLHT5	LOC_Os04g47420	1921	-	Sp1	GGGCGG	light responsive element
OsLHT5	LOC_Os04g47420	1942	-	Sp1	GGGCGG	light responsive element
OsLHT5	LOC_Os04g47420	540	-	TGACG-motif	TGACG	cis-acting regulatory element involved in the MeJA-responsiveness

OsLHT5	LOC_Os04g47420	784	+	TGACG-motif	TGACG	cis-acting regulatory element involved in the MeJA-responsiveness
OsLHT5	LOC_Os04g47420	969	-	TGACG-motif	TGACG	cis-acting regulatory element involved in the MeJA-responsiveness
OsLHT5	LOC_Os04g47420	1087	-	TGACG-motif	TGACG	cis-acting regulatory element involved in the MeJA-responsiveness
OsLHT5	LOC_Os04g47420	1717	+	CAT-box	GCCACT	cis-acting regulatory element related to meristem expression
OsLHT5	LOC_Os04g47420	1376	-	ACE	GACACGTATG	cis-acting element involved in light responsiveness
OsLHT5	LOC_Os04g47420	164	+	TATA-box	TATA	core promoter element around -30 of transcription start
OsLHT5	LOC_Os04g47420	338	+	TATA-box	ATATAT	core promoter element around -30 of transcription start
OsLHT5	LOC_Os04g47420	339	+	TATA-box	TATA	core promoter element around -30 of transcription start
OsLHT5	LOC_Os04g47420	476	+	TATA-box	TATA	core promoter element around -30 of transcription start
OsLHT5	LOC_Os04g47420	602	+	TATA-box	TATA	core promoter element around -30 of transcription start
OsLHT5	LOC_Os04g47420	1017	-	TATA-box	TATA	core promoter element around -30 of transcription start
OsLHT5	LOC_Os04g47420	1536	-	TATA-box	TATAAGAA	core promoter element around -30 of transcription start
OsLHT5	LOC_Os04g47420	1539	-	TATA-box	TATAA	core promoter element around -30 of transcription start
OsLHT5	LOC_Os04g47420	1540	-	TATA-box	TATA	core promoter element around -30 of transcription start
OsLHT5	LOC_Os04g47420	1653	-	TATA-box	TATA	core promoter element around -30 of transcription start
OsLHT5	LOC_Os04g47420	1877	-	TATA-box	TATA	core promoter element around -30 of transcription start
OsLHT5	LOC_Os04g47420	298	+	A-box	CCGTCC	cis-acting regulatory element

OsLHT5	LOC_Os04g47420	839	-	A-box	CCGTCC	cis-acting regulatory element
OsLHT5	LOC_Os04g47420	1639	+	A-box	CCGTCC	cis-acting regulatory element
OsLHT5	LOC_Os04g47420	681	-	chs-CMA2a	TCACTTGA	part of a light responsive element
OsLHT6	LOC_Os12g30040	326	-	Sp1	GGGCGG	light responsive element
OsLHT6	LOC_Os12g30040	556	+	Sp1	GGGCGG	light responsive element
OsLHT6	LOC_Os12g30040	1005	-	Sp1	GGGCGG	light responsive element
OsLHT6	LOC_Os12g30040	1193	+	Sp1	GGGCGG	light responsive element
OsLHT6	LOC_Os12g30040	1718	-	Sp1	GGGCGG	light responsive element
OsLHT6	LOC_Os12g30040	426	+	ARE	AAACCA	cis-acting regulatory element essential for the anaerobic induction
OsLHT6	LOC_Os12g30040	342	+	GC-motif	CCCCCG	enhancer-like element involved in anoxic specific inducibility
OsLHT6	LOC_Os12g30040	382	+	GC-motif	CCCCCG	enhancer-like element involved in anoxic specific inducibility
OsLHT6	LOC_Os12g30040	743	-	GC-motif	CCCCCG	enhancer-like element involved in anoxic specific inducibility
OsLHT6	LOC_Os12g30040	1865	+	CGTCA-motif	CGTCA	cis-acting regulatory element involved in the MeJA-responsiveness
OsLHT6	LOC_Os12g30040	207	+	CAT-box	GCCACT	cis-acting regulatory element related to meristem expression
OsLHT6	LOC_Os12g30040	1582	+	CAT-box	GCCACT	cis-acting regulatory element related to meristem expression
OsLHT6	LOC_Os12g30040	1268	-	MBS	CAACTG	MYB binding site involved in drought-inducibility
OsLHT6	LOC_Os12g30040	603	-	LTR	CCGAAA	cis-acting element involved in low-temperature responsiveness
OsLHT6	LOC_Os12g30040	1627	+	LTR	CCGAAA	cis-acting element involved in low-temperature responsiveness
OsLHT6	LOC_Os12g30040	7	+	CAAT-box	CAAT	common cis-acting element in promoter and enhancer regions

OsLHT6	LOC_Os12g30040	150	-	CAAT-box	CAAAT	common cis-acting element in promoter and enhancer regions
OsLHT6	LOC_Os12g30040	202	+	CAAT-box	CAAT	common cis-acting element in promoter and enhancer regions
OsLHT6	LOC_Os12g30040	204	-	CAAT-box	CAAT	common cis-acting element in promoter and enhancer regions
OsLHT6	LOC_Os12g30040	234	-	CAAT-box	CAAAT	common cis-acting element in promoter and enhancer regions
OsLHT6	LOC_Os12g30040	330	+	CAAT-box	CCAAT	common cis-acting element in promoter and enhancer regions
OsLHT6	LOC_Os12g30040	331	+	CAAT-box	CAAT	common cis-acting element in promoter and enhancer regions
OsLHT6	LOC_Os12g30040	450	-	CAAT-box	CCAAT	common cis-acting element in promoter and enhancer regions
OsLHT6	LOC_Os12g30040	459	-	CAAT-box	CAAT	common cis-acting element in promoter and enhancer regions
OsLHT6	LOC_Os12g30040	952	-	CAAT-box	CAAAT	common cis-acting element in promoter and enhancer regions
OsLHT6	LOC_Os12g30040	1216	-	CAAT-box	CAAAT	common cis-acting element in promoter and enhancer regions
OsLHT6	LOC_Os12g30040	1354	-	CAAT-box	CAAAT	common cis-acting element in promoter and enhancer regions
OsLHT6	LOC_Os12g30040	1376	+	CAAT-box	CAAAT	common cis-acting element in promoter and enhancer regions
OsLHT6	LOC_Os12g30040	1381	-	CAAT-box	CAAT	common cis-acting element in promoter and enhancer regions
OsLHT6	LOC_Os12g30040	1385	-	CAAT-box	CAAT	common cis-acting element in promoter and enhancer regions
OsLHT6	LOC_Os12g30040	1413	-	CAAT-box	CAAT	common cis-acting element in promoter and enhancer regions
OsLHT6	LOC_Os12g30040	1422	-	CAAT-box	CCAAT	common cis-acting element in promoter and enhancer regions
OsLHT6	LOC_Os12g30040	1822	-	CAAT-box	CAAT	common cis-acting element in promoter and enhancer regions
OsLHT6	LOC_Os12g30040	1860	-	CAAT-box	CAAT	common cis-acting element in promoter and enhancer regions
OsLHT6	LOC_Os12g30040	1735	+	A-box	CCGTCC	cis-acting regulatory element
OsLHT6	LOC_Os12g30040	42	+	TATA-box	TATA	core promoter element around -30 of transcription start

OsLHT6	LOC_Os12g30040	67	-	TATA-box	TATAAAT	core promoter element around -30 of transcription start
OsLHT6	LOC_Os12g30040	68	-	TATA-box	TATAAA	core promoter element around -30 of transcription start
OsLHT6	LOC_Os12g30040	69	-	TATA-box	TATAA	core promoter element around -30 of transcription start
OsLHT6	LOC_Os12g30040	70	+	TATA-box	TATA	core promoter element around -30 of transcription start
OsLHT6	LOC_Os12g30040	170	+	TATA-box	ATATAA	core promoter element around -30 of transcription start
OsLHT6	LOC_Os12g30040	171	+	TATA-box	TATA	core promoter element around -30 of transcription start
OsLHT6	LOC_Os12g30040	192	+	TATA-box	TATA	core promoter element around -30 of transcription start
OsLHT6	LOC_Os12g30040	827	-	TATA-box	TATACA	core promoter element around -30 of transcription start
OsLHT6	LOC_Os12g30040	829	+	TATA-box	TATA	core promoter element around -30 of transcription start
OsLHT6	LOC_Os12g30040	911	+	TATA-box	TATTTAAA	core promoter element around -30 of transcription start
OsLHT6	LOC_Os12g30040	1321	-	TATA-box	TATATA	core promoter element around -30 of transcription start
OsLHT6	LOC_Os12g30040	1323	-	TATA-box	TATA	core promoter element around -30 of transcription start
OsLHT6	LOC_Os12g30040	1328	-	TATA-box	TATAAAT	core promoter element around -30 of transcription start
OsLHT6	LOC_Os12g30040	1329	-	TATA-box	TATAAA	core promoter element around -30 of transcription start
OsLHT6	LOC_Os12g30040	1330	-	TATA-box	TATAA	core promoter element around -30 of transcription start
OsLHT6	LOC_Os12g30040	1331	-	TATA-box	TATA	core promoter element around -30 of transcription start
OsLHT6	LOC_Os12g30040	1359	-	TATA-box	TATACA	core promoter element around -30 of transcription start
OsLHT6	LOC_Os12g30040	1361	-	TATA-box	TATA	core promoter element around -30 of transcription start
OsLHT6	LOC_Os12g30040	1394	-	TATA-box	TATATA	core promoter element around -30 of transcription start
OsLHT6	LOC_Os12g30040	1395	-	TATA-box	ATATAT	core promoter element around -30 of transcription start

OsLHT6	LOC_Os12g30040	1396	-	TATA-box	TATA	core promoter element around -30 of transcription start
OsLHT6	LOC_Os12g30040	1398	-	TATA-box	TATAAATA	core promoter element around -30 of transcription start
OsLHT6	LOC_Os12g30040	1399	-	TATA-box	TATAAAT	core promoter element around -30 of transcription start
OsLHT6	LOC_Os12g30040	1400	-	TATA-box	TATAAA	core promoter element around -30 of transcription start
OsLHT6	LOC_Os12g30040	1401	-	TATA-box	TATAA	core promoter element around -30 of transcription start
OsLHT6	LOC_Os12g30040	1402	-	TATA-box	TATA	core promoter element around -30 of transcription start
OsLHT6	LOC_Os12g30040	657	+	G-Box	CACGTG	cis-acting regulatory element involved in light responsiveness
OsLHT6	LOC_Os12g30040	1600	-	G-Box	CACGTG	cis-acting regulatory element involved in light responsiveness
OsLHT6	LOC_Os12g30040	657	+	G-box	CACGTG	cis-acting regulatory element involved in light responsiveness
OsLHT6	LOC_Os12g30040	1525	-	G-box	CACGTC	cis-acting regulatory element involved in light responsiveness
OsLHT6	LOC_Os12g30040	1600	-	G-box	CACGTG	cis-acting regulatory element involved in light responsiveness
OsLHT6	LOC_Os12g30040	1666	+	G-box	CACGTC	cis-acting regulatory element involved in light responsiveness
OsLHT6	LOC_Os12g30040	1915	+	G-box	CACGAC	cis-acting regulatory element involved in light responsiveness
OsLHT6	LOC_Os12g30040	1834	+	TCCC-motif	TCTCCCT	part of a light responsive element
OsLHT6	LOC_Os12g30040	1928	+	TCCC-motif	TCTCCCT	part of a light responsive element
OsLHT6	LOC_Os12g30040	1940	-	I-box	gGATAAGGTG	part of a light responsive element
OsLHT6	LOC_Os12g30040	1865	-	TGACG-motif	TGACG	cis-acting regulatory element involved in the MeJA-responsiveness
OsLHT6	LOC_Os12g30040	657	+	ABRE	CACGTG	cis-acting element involved in the abscisic acid responsiveness
OsLHT6	LOC_Os12g30040	658	+	ABRE	ACGTG	cis-acting element involved in the abscisic acid responsiveness

OsLHT6	LOC_Os12g30040	1526	+	ABRE	ACGTG	cis-acting element involved in the abscisic acid responsiveness
OsLHT6	LOC_Os12g30040	1598	-	ABRE	CGCACGTGTC	cis-acting element involved in the abscisic acid responsiveness
OsLHT6	LOC_Os12g30040	1600	-	ABRE	CACGTG	cis-acting element involved in the abscisic acid responsiveness
OsLHT6	LOC_Os12g30040	1601	+	ABRE	ACGTG	cis-acting element involved in the abscisic acid responsiveness
OsLHT6	LOC_Os12g30040	1666	-	ABRE	ACGTG	cis-acting element involved in the abscisic acid responsiveness
